# Interaction Networks Are Driven by Community-Responsive Phenotypes in a Chitin-Degrading Consortium of Soil Microbes

**DOI:** 10.1128/msystems.00372-22

**Published:** 2022-09-26

**Authors:** Ryan McClure, Yuliya Farris, Robert Danczak, William Nelson, Hyun-Seob Song, Aimee Kessell, Joon-Yong Lee, Sneha Couvillion, Christopher Henry, Janet K. Jansson, Kirsten S. Hofmockel

**Affiliations:** a Biological Sciences Division, Pacific Northwest National Laboratory, Richland, Washington, USA; b Department of Agronomy, Iowa State University, Ames, Iowa, USA; c Department of Biological Systems Engineering, University of Nebraska—Lincoln, Lincoln, Nebraska, USA; d Department of Food Science and Technology, Nebraska Food for Health Center, University of Nebraska—Lincoln, Lincoln, Nebraska, USA; e Data Science and Learning Division, Argonne National Laboratory, Lemont, Illinois, USA; University of Massachusetts Amherst

**Keywords:** soil microbiome, consortia, model microbiome, chitin decomposition, metabolic interactions, chitin

## Abstract

Soil microorganisms provide key ecological functions that often rely on metabolic interactions between individual populations of the soil microbiome. To better understand these interactions and community processes, we used chitin, a major carbon and nitrogen source in soil, as a test substrate to investigate microbial interactions during its decomposition. Chitin was applied to a model soil consortium that we developed, “model soil consortium-2” (MSC-2), consisting of eight members of diverse phyla and including both chitin degraders and nondegraders. A multiomics approach revealed how MSC-2 community-level processes during chitin decomposition differ from monocultures of the constituent species. Emergent properties of both species and the community were found, including changes in the chitin degradation potential of *Streptomyces* species and organization of all species into distinct roles in the chitin degradation process. The members of MSC-2 were further evaluated via metatranscriptomics and community metabolomics. Intriguingly, the most abundant members of MSC-2 were not those that were able to metabolize chitin itself, but rather those that were able to take full advantage of interspecies interactions to grow on chitin decomposition products. Using a model soil consortium greatly increased our knowledge of how carbon is decomposed and metabolized in a community setting, showing that niche size, rather than species metabolic capacity, can drive success and that certain species become active carbon degraders only in the context of their surrounding community. These conclusions fill important knowledge gaps that are key to our understanding of community interactions that support carbon and nitrogen cycling in soil.

**IMPORTANCE** The soil microbiome performs many functions that are key to ecology, agriculture, and nutrient cycling. However, because of the complexity of this ecosystem we do not know the molecular details of the interactions between microbial species that lead to these important functions. Here, we use a representative but simplified model community of bacteria to understand the details of these interactions. We show that certain species act as primary degraders of carbon sources and that the most successful species are likely those that can take the most advantage of breakdown products, not necessarily the primary degraders. We also show that a species phenotype, including whether it is a primary degrader or not, is driven in large part by the membership of the community it resides in. These conclusions are critical to a better understanding of the soil microbial interaction network and how these interactions drive central soil microbiome functions.

## INTRODUCTION

The soil microbiome carries out several important ecological functions, including carbon (C) and nitrogen (N) cycling and plant growth promotion (1–3). Central to these functions are interactions between the species that comprise soil microbial communities (4–6). While the combined genomic and metabolic potential of the individual species of the soil microbiome is vast, novel functions can emerge at the community level through metabolic interactions (7). A better understanding of these interactions will lead to a more complete view of the constituent organism’s and community’s functional capacity and will greatly expand our knowledge of how interaction networks can be affected by nutrient or environmental shifts (8, 9).

One of the most enigmatic microbial functions that depends on species interactions is the decomposition of soil organic matter. Identification of metabolic interactions involved in decomposition is particularly challenging due to the complexity of organic substrates in soil, the biodiversity of organisms involved, and difficulty in extracting samples at microbiological scales in soil (10). As a result, few studies have interrogated the taxon-specific gene expression and community metabolism that occur during C decomposition in soil. Microbially driven breakdown of plant-derived matter, such as cellulose (11, 12), is of great interest due to its ubiquity in soil environments. While the genomic potential for cellulase enzyme production has been detected in almost 40% of bacterial genomes in the Carbohydrate-Active Enzyme database, only a small number of organisms have been shown to digest cellulose independently in pure culture (13). The challenges described above in soil have made interactions centered on breakdown of cellulose difficult to ascertain. Even less is known about the decomposition of additional abundant molecules such as chitin that contribute to both carbon and nitrogen cycling in soil (14, 15). While chitinases, like cellulases, are widespread in bacteria (16), their expression is not universal and is differentially controlled by different species or even within a population (17). Previous studies using fluorescent reporter assays focused on both transcripts and proteins showed that only a subpopulation of cells in a pure culture of a chitin-degrading strain actually produce chitinases, with the remaining cells feeding off breakdown products (18, 19). In addition, chitinases are often not cell associated, meaning breakdown products are available not only to the species expressing the chitinase but also to other species of local community as well. As such, there is a strong element of inter/intraspecies interactions centered on community metabolism of chitin and its decomposition products.

The molecular details of community interactions during chitin decomposition and the generation of breakdown products have been previously studied in some detail in non-soil environments, especially in aquatic systems (16, 20). Chitin is the polymer of (1→4)-β-linked *N-*acetyl-d-glucosamine (NAG) monomers, and the decomposition of chitin into its NAG monomers is driven either by membrane-bound chitinases or through excreted non-cell-associated enzymes. Once converted into NAG oligomers, import and intracellular metabolism are possible and dimers are converted to monomers via β-*N*-acetylglucosaminidases. Numerous species have been found that contain transporters for uptake of NAG molecules or metabolic genes that act on NAG without a corresponding set of chitin-degrading genes, suggesting that some bacteria rely on NAG produced through chitin breakdown carried out by other species in their neighborhood (21). In supporting chitin degraders, it is possible that NAG consumers contribute to more effective chitin breakdown by removing downstream metabolites to increase enzyme efficiency (22) or providing additional metabolic benefits such as vitamins to alleviate the energetic costs expended by chitinase producers (23). As a result, growth promotion is driven by chitin degraders and secondary consumers through metabolic cross talk, where growth of constituents is maximized through cooperation.

Ecological theory suggests that the ability of a species to take advantage of exometabolites for growth is driven in part by the size of a species’ fundamental niche (C sources they can metabolize themselves) compared to the realized niche (C sources that can be metabolized by the complete community) (24). A large fundamental niche indicates that species may be able to take advantage of the presence of many exometabolites independently of other species. Yet it is not clear who has an advantage during growth with chitin as the sole carbon source—chitin degraders or consumers—nor is it clear how this advantage may be related to a species’ fundamental niche size. Laboratory growth experiments have suggested that primary consumers of complex carbon sources do not have a growth advantage (25), but whether this is a consistent rule and how this extends to field environments is not known.

Finally, the identity of chitin degraders or consumers may shift in response to community dynamics because species can express emergent properties with other species that they do not express during growth alone. For example, previous work from our group explored interactions centered on chitin breakdown by investigating a naturally evolved community of soil microbes developed using chitin as the major C and N source (26). Analyses of this community, MSC-1 (model soil consortium-1), identified several species, primarily a species of *Rhodococcus*, that occupied central positions in a 16S rRNA gene amplicon coabundance network, suggesting that they may be dominant chitin degraders and in turn provide assimilable substrates to other community members. Following isolation of specific constituents of MSC-1, subsequent coculture work revealed that several genera of this community (including *Ensifer*, *Dyadobacter*, and *Rhizobium* grown with *Rhodococcus* and *Streptomyces* grown with *Ensifer*) showed higher biomass when cocultured versus in monoculture. These initial studies suggest a network of interactions centered on chitin breakdown. However, there is a knowledge gap regarding the molecular details of how a community of chitin degraders and nondegraders organize themselves to break down chitin and share metabolic products.

Here, we aimed to fill these knowledge gaps by constructing and examining a new model community assembled from MSC-1 isolates, model soil consortium-2 (MSC-2). To delineate how MSC-2 degrades chitin, we used a multiomics approach combining species abundance analyses and expressed functions (metatranscriptomics) and extracellular nutrient pools (metabolomics) applied to chitin growth assays of the MSC-2 consortium as a community as well as its constituents in monoculture. We set out to answer three questions. (i) Which species have a growth advantage in a community—chitin degraders or species with large fundamental niches that can take the most advantage of chitin breakdown products? (ii) To what degree do MSC-2 members organize themselves into a chitin-degrading community, with each member contributing certain aspects of the breakdown process (cleaving of chitin polymer bonds, breakdown of NAG trimers or dimers, and processing of NAG monomers into further C and N pathways)? Finally, with an eye on native soil ecology, (iii) to what extent is the chitin decomposition phenotype of a species defined by the composition of the community in which this species grows? Answering these questions will shed light on how diverse bacterial species contribute to community decomposition and to what degree the ability to degrade organic C and/or assimilate breakdown products translates into a growth advantage in a mixed community.

## RESULTS

### Development of MSC-2 to investigate community and constituent roles in chitin degradation.

**FIG 1:**
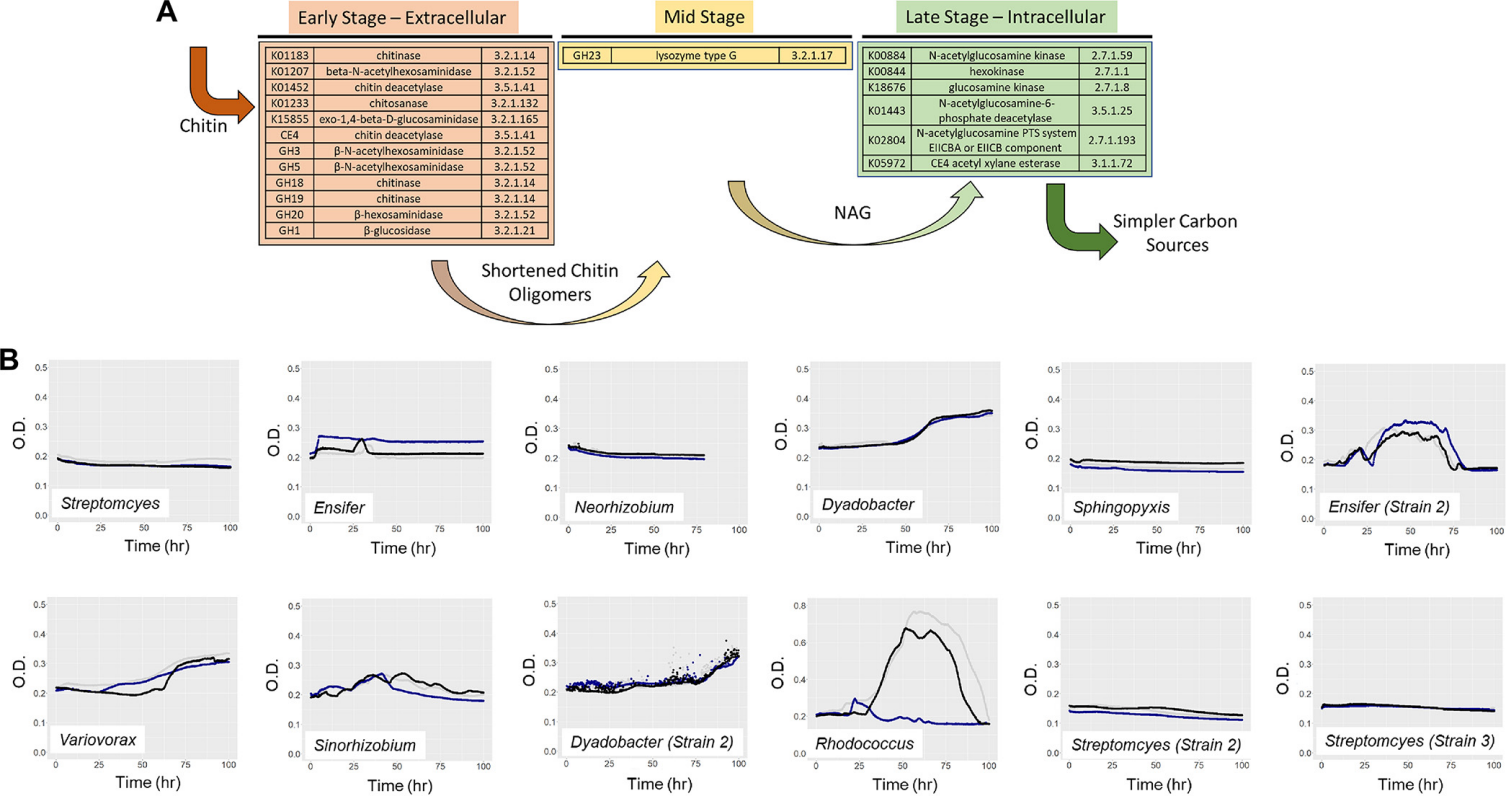
Chitin breakdown pathway and growth of MSC-1 isolates. (A) The known chitin breakdown pathway is illustrated with glycosyl hydrolase names, KO numbers, and/or EC numbers assigned to early, mid, and late stages of chitin metabolism. (B) Growth curves for each MSC-2 strain are shown with time (in hours) on the *x* axis and optical density at 600 nm (OD_600_) on the *y* axis. Three biological replicates were performed for each strain, indicated by blue, black, and gray lines. Note the *y* axis scale for *Rhodococcus* differs from those of other panels due to high biomass production.

Our previous work described the development and analysis of a naturally evolved model microbial consortium of ~35 species (model soil consortium-1 [MSC-1]) (26). Here, we first obtained new isolates from this community by plating onto agar media and validated isolate identities by sequencing. As a result, members of the *Sphingopyxis* and *Sinorhizobium* genera were isolated, as well as novel strains of the *Ensifer*, *Dyadobacter*, and *Streptomyces* genera (see Table S1 in the supplemental material). As MSC-1 was originally isolated by enrichment under high-chitin conditions, we aimed to determine if any of the MSC-1 isolates could grow using chitin as the sole source of C (Fig. 1) under well-mixed liquid cultivation conditions. This analysis showed that *Dyadobacter*, one *Ensifer* strain (strain PNNL_MSC-1_Str_011), *Variovorax*, *Sinorhizobium*, and *Rhodococcus* grew with chitin, while the remaining strains of *Streptomyces*, *Ensifer*, *Neorhizobium*, and *Sphingopyxis* did not (Fig. 1B). We also tested for growth of *Streptomyces*, *Neorhizobium*, and *Sphingopyxis* on larger amounts of chitin (500 and 1,000 ppm) and observed similar lack of growth (data not shown). Comparisons of the *Ensifer* strains that did and did not grow on chitin showed some minor sequence differences but no apparent differences in annotated pathways. These results lay out the monoculture phenotypes of these species and allow for future comparisons of isolate phenotypes to emergent phenotypes expressed during community growth. From this set of 12 species, we selected eight that would comprise microbial soil consortium-2 (MSC-2) (Table 1). Selection was carried out with the goals of (i) including both chitin degraders and nondegraders and (ii) maximizing taxonomic diversity in MSC-2. Subsequent discussion of MSC-2 is defined as these eight species cultured together.

**TABLE 1:** Taxonomy, phenotype, and genomic information on MSC-2 members

Isolate designation	GTDB taxonomy^*a*^	No. of genes	No. of unique genes	Chitin growth	Max OD_600_^*b*^	Max OD SD	Time to reach Max OD (h)	Chitinase genes^*c*^	SOX system
PNNL_MSC-1_Str_001	*Streptomyces* sp001905665	3,029	14	No	0.197	0.007	NA^*d*^	(7/8) mannan endo-1 (GH5) (K01218), β-glucosidase (GH3) (EC 3.2.1.21), hexosaminidase (GH20) (EC 3.2.1.52), β-*N*-acetylhexosaminidase (GH3) (EC 3.2.1.52), chitin-binding protein (K03933), chitinase (GH18) (EC 3.2.1.14), β-glucosidase (GH3) (EC 3.2.1.21), peptidoglycan-*N*-acetylglucosamine deacetylase (CE4) (EC 3.5.1.104), endoglucanase (GH5) (EC 3.2.1.4), *N*-acetylglucosamine-6-phosphate deacetylase (EC 3.5.1.25), *N*-acetylglucosamine PTS system EIICBA or EIICB component (EC 2.7.1.193)	No
PNNL_MSC-1_Str_005	*Neorhizobium tomejilense*	3,619	48	No	0.183	0.011	NA	(6/8) soluble lytic murein transglycosylase (GH23) (EC 4.2.2.−), β-*N*-acetylhexosaminidase (GH3) (EC 3.2.1.52), endoglucanase (GH5) (EC 3.2.1.4), putative chitinase (GH19) (K03791), *N*-acetylglucosamine-6-phosphate deacetylase (EC 3.5.1.25), glucosamine kinase (EC 2.7.1.8)	Yes
PNNL_MSC-1_Str_007	Dyadobacter fermentans	2,270	30	Yes	0.351	0.008	99	(5/8) membrane-bound lytic murein transglycosylase D (GH23) (EC 4.2.2.−), β-*N*-acetylhexosaminidase (GH3) (EC 3.2.1.52), hexosaminidase (GH20) (EC 3.2.1.52), chitinase (GH18) (EC 3.2.1.14), endoglucanase (GH5) (EC 3.2.1.4), *N*-acetylglucosamine-6-phosphate deacetylase (EC 3.5.1.25), chitosanase (EC 3.2.1.132)	Yes
PNNL_MSC-1_Str_008	Sphingopyxis fribergensis	2,257	62	No	0.188	0.008	NA	(4/8) soluble lytic murein transglycosylase (GH23) (EC 4.2.2.−), chitinase (GH18) (EC 3.2.1.14), hexosaminidase (GH20) (EC 3.2.1.52), β-glucosidase (GH3) (EC 3.2.1.21), *N*-acetylglucosamine PTS system EIICBA or EIICB component (EC 2.7.1.193), *N*-acetylglucosamine-6-phosphate deacetylase (EC 3.5.1.25)	No
PNNL_MSC-1_Str_011	Ensifer adhaerens	3,978	26	Yes	0.289	0.022	35	(6/8) membrane-bound lytic murein transglycosylase F (GH23) (EC 4.2.2.−), soluble lytic murein transglycosylase (GH23) (EC 4.2.2.−), β-*N*-acetylhexosaminidase (GH3) (EC 3.2.1.52), putative chitinase (GH19) (K03791), hexosaminidase (GH20) (EC 3.2.1.52), *N*-acetylglucosamine-6-phosphate deacetylase (EC 3.5.1.25), glucosamine kinase (EC 2.7.1.8)	No
PNNL_MSC-1_Str_012	Variovorax beijingensis	3,996	0	Yes	0.322	0.015	109	(3/8) peptidoglycan-*N*-acetylglucosamine deacetylase (CE4) (EC 3.5.1.104), β-glucosidase (GH3) (EC 3.2.1.21), membrane-bound lytic murein transglycosylase D (GH23) (EC 4.2.2.−), β-*N*-acetylhexosaminidase (GH3) (EC 3.2.1.52), soluble lytic murein transglycosylase (GH23) (EC 4.2.2.−), poly-β-1,6-*N*-acetyl-d-glucosamine *N*-deacetylase (CE4) (EC 3.5.1.−)	No
PNNL_MSC-1_Str_014	Sinorhizobium meliloti	3,535	50	Yes	0.259	0.011	42	(7/8) soluble lytic murein transglycosylase (GH23) (EC 4.2.2.−), putative chitinase (GH19) (K03791), β-*N*-acetylhexosaminidase (GH3) (EC 3.2.1.52), hexosaminidase (GH20) (EC 3.2.1.52), glucosamine kinase (EC 2.7.1.8), *N*-acetylglucosamine-6-phosphate deacetylase (EC 3.5.1.25)	Yes
PNNL_MSC-1_Str_016	*Rhodococcus* sp003130705	3,602	210	Yes	0.697	0.101	78	(3/8) mannan endo-1,4-β-mannosidase (GH5) (EC 3.2.1.78), peptidoglycan-*N*-acetylglucosamine deacetylase (CE4) (EC 3.5.1.104), β-*N*-acetylhexosaminidase (GH3) (EC 3.2.1.52)	No

a GTDB, Genome Taxonomy Database.

b Maximum (Max) optical density at 600 nm for each species when grown on chitin. Results are the average from three replicates.

c The numbers in parentheses indicate how many chitin metabolism genes this species contained out of eight included.

d NA, not applicable (no growth apparent).

10.1128/msystems.00372-22.7 TABLE S1 List of species isolated from MSC-1. Download Table S1, PDF file, 0.1 MB. Copyright © 2022 McClure et al. 2022 McClure et al. https://creativecommons.org/licenses/by/4.0/ This content is distributed under the terms of the Creative Commons Attribution 4.0 International license.

### Measuring fundamental niche size of MSC-2 members.

**FIG 2:**
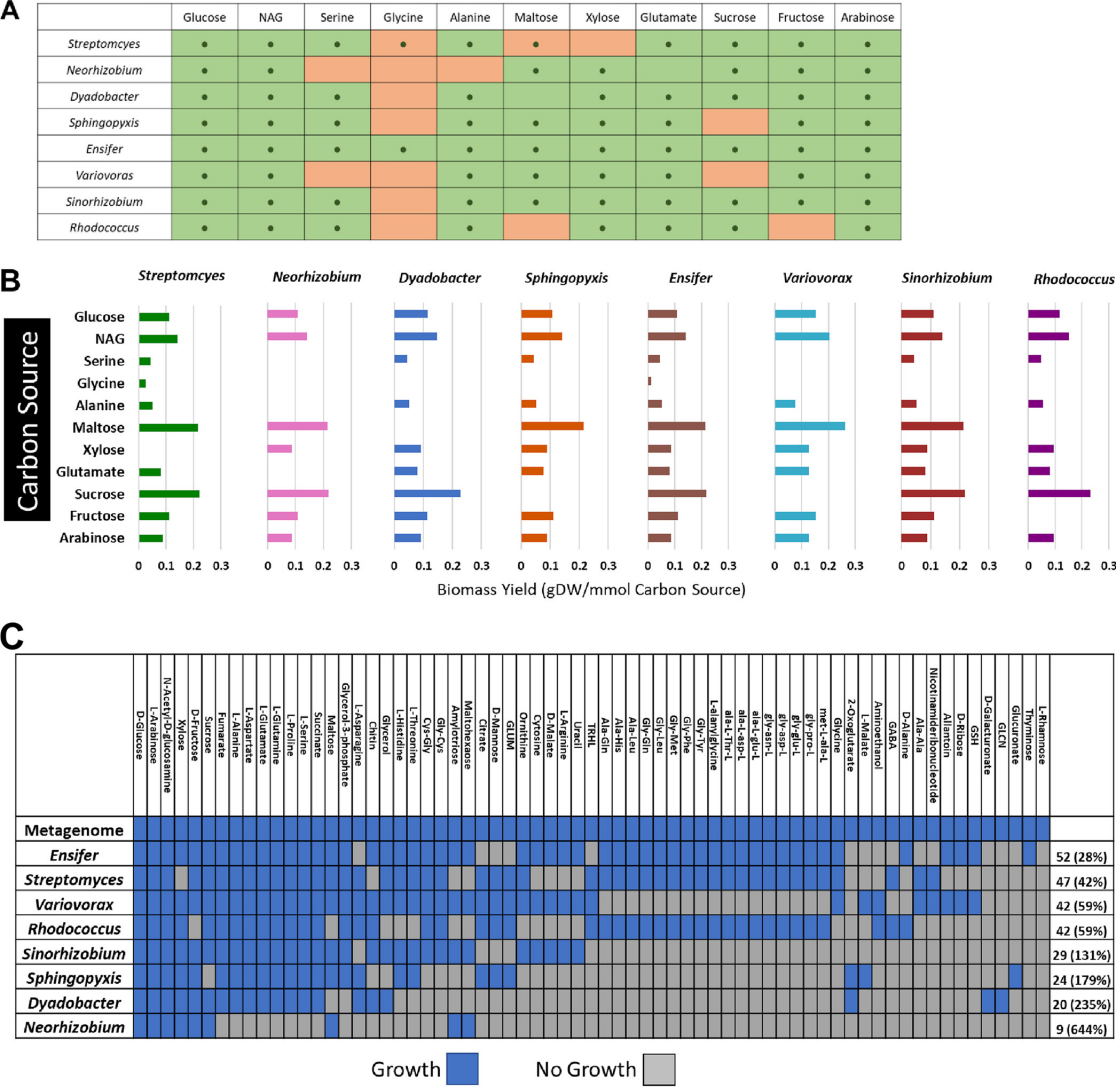
Growth of MSC-2 constituents on variable C/N sources. (A) Growth of strains with various carbon sources. Panel cells with a dot indicate that the metabolic model predicted growth for that species on that C source. Cells with no dot indicate there was no predicted growth for that species on that C source. Green cells indicate the species was experimentally confirmed to grow under that C source. Orange cells indicate that the species was experimentally confirmed to not grow under that C source. (B) Predicted biomass production (grams of dry weight [gDW]) for each species when grown in isolation with each carbon source, using flux balance analysis (FBA). (C) Fundamental and realized niches of each of the species of MSC-2. Blue squares indicate predicted or confirmed growth on the carbon source, and gray squares indicate predicted or confirmed lack of growth. Member species rows are ranked from largest to smallest fundamental niche. C source columns are ranked from most used to least used C sources. Numbers at the ends of the rows indicate the size of the fundamental niche, with number in parentheses indicating increase in size of the realized niche (complete metagenome niche size) compared to the fundamental niche.

As a major goal of this study was to demonstrate the power of model communities to test ecological theory, we next carried out experiments using MSC-2 to test how fundamental niche size affects success, as measured by relative abundance, in a community. We assessed niche size by measuring the growth of each species under a variety of C sources as described in Materials and Methods (Fig. 2). While many of the tested substrates could be potential C and/or N sources, all experiments were carried out using the substrates as the sole C source and with ammonium added as a source of N. In certain cases, this led to a change in C/N ratios for some substrates. Because the ability of MSC-2 species to use these substrates as a source of C was the focus of these experiments, all substrates were added at the same concentration (10 mM).

All MSC-2 species were able to use glucose, NAG, glutamate, or arabinose as the sole C source, and all species except *Neorhizobium*, *Streptomyces*, and *Rhodococcus* were able to use alanine, xylose, and fructose, respectively, as well (Fig. 2A). Only *Ensifer* was able to use glycine as a substrate. Growth on other C and N sources was mixed, but generally most strains were able to use most nutrient sources.

With this information, we next generated metabolic models for each of the eight MSC-2 species using the Department of Energy Systems Biology Knowledgebase (KBase) (27) and simulated the biomass production of each isolate grown axenically on the suite of carbon sources using flux balance analysis (FBA) (Fig. 2B). With this analysis, the biomass yields on NAG for all species were larger than those on glucose, fructose, arabinose, and several amino acids. Similarly, for those that grew on disaccharides, maltose, and sucrose, the biomass yield per millimole of C source was almost double that predicted when grown on glucose and was also higher than that on NAG. Amino acids in general led to lower predicted biomass yields for MSC-2 species than sugars. This may be an effect of these amino acids also acting as an N source in experiments where an abundant N source (ammonia) was also present, possibly leading to altered C/N ratios that may affect growth as has been seen previously (28, 29). This was one of main reasons we chose chitin in subsequent experiments, to investigate a coupled C and N source. All of the members of MSC-2 had high yields on the main C breakdown product of chitin (NAG) but also responded well to other C sources, showing that while they are well suited to a high-chitin environment, they have pathways to metabolize several other C sources that are prevalent in soil.

The predicted growth patterns of the MSC-2 species were used to infer their fundamental and realized niches (24) (Fig. 2C). Fundamental niches are defined as the C sources that an MSC-2 species is predicted to metabolize itself (i.e., when grown in monoculture). The realized niche is the set of carbon sources that can be metabolized by the community as a whole, the sum of each specie’s fundamental niche. This analysis revealed that *Ensifer* had the largest fundamental niche followed by *Streptomyces*, *Variovorax*, and *Rhodococcus*. In contrast, *Neorhizobium* and *Dyadobacter* had the smallest fundamental niches. This suggests that *Ensifer* can take the most advantage of interactions resulting from residing in the MSC-2 community, compared to monoculture growth on a single C source, due to its larger fundamental niche; species with smaller niches may not be able to benefit as much from metabolic products resulting from community growth.

### Genomic analysis of MSC-2 members.

**FIG 3:**
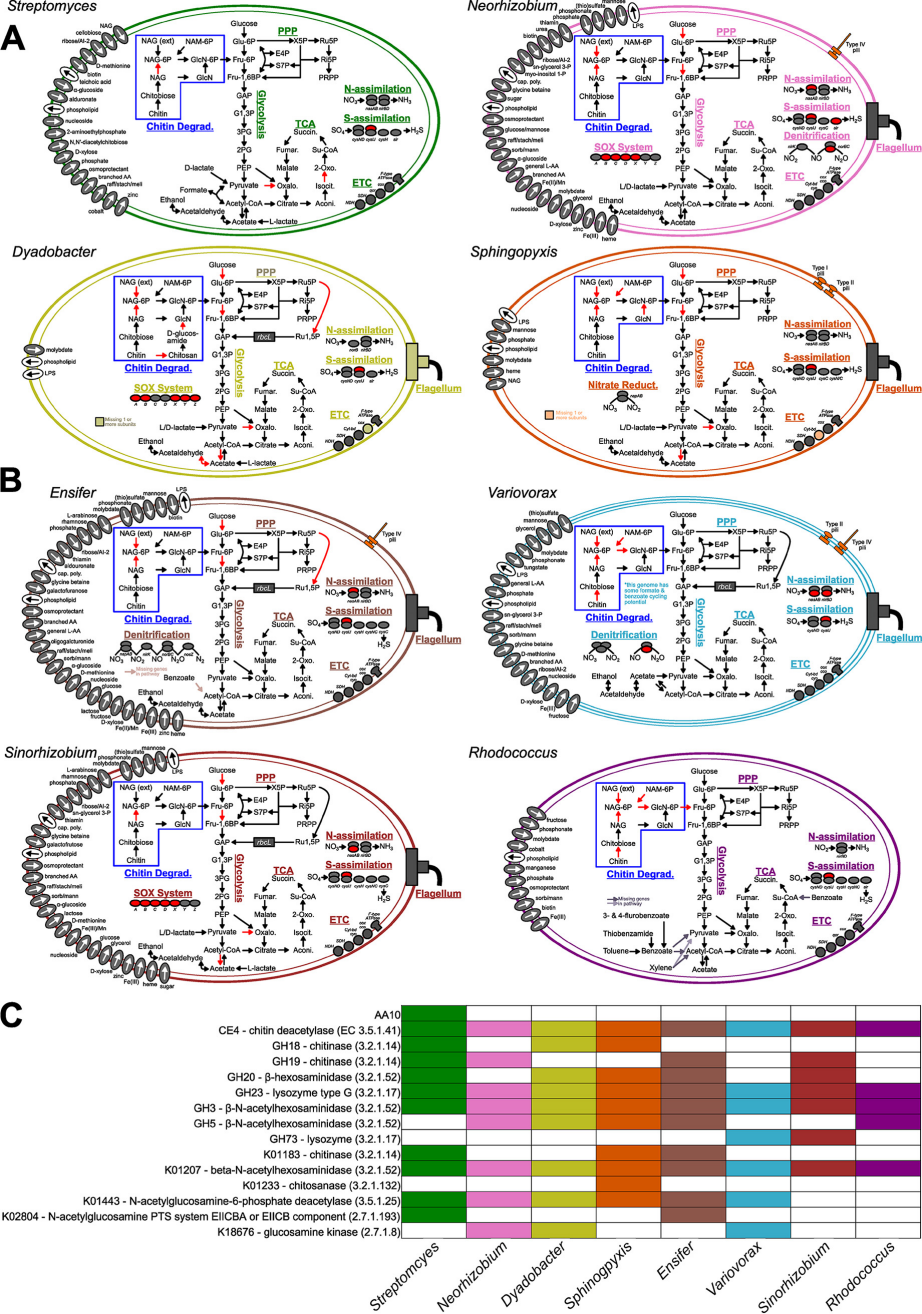
Genomic potential of MSC-2 constituents. (A) *Streptomyces*, *Neorhizobium*, *Dyadobacter*, and *Sphingopyxis* strains are shown. Genes predicted to encode importers and exporters are indicated by arrows and gray or white circles, respectively. Chitin degradation pathways are highlighted in the blue boxes, and pilin or flagellar systems are indicated on the edges. For chitin pathways, black arrows indicate known reactions that the species is predicted to encode and red arrows indicate known reactions that the species is not predicted to encode. (B) *Ensifer*, *Variovorax*, *Sinorhizobium*, and *Rhodococcus* are shown. (C) Heat map showing prevalence of genes involved in chitin/NAG metabolism in MSC-2 constituents. The color scheme matches panels A and B, and colored cells indicate the presence of a gene.

Whole-genome sequencing was used to identify MSC-2 members to the greatest taxonomic depth possible and to gather information on their metabolic potential. Sequencing results and taxonomic and strain information are shown in Table 1. All MSC-2 members were predicted to contain at least some genes involved in chitin degradation, but the specific genes that each member contained varied (Fig. 3 and Table 1). Coverage of the chitin breakdown pathway was defined by having a certain number of the eight genes we assigned to the chitin/NAG breakdown and assimilation pathway. In addition, several other central processes (sulfur [S] and N assimilation, denitrification, mobility from flagellar genes, and secretion systems) varied among MSC-2 members (Fig. 3). Specifically, we observed substantial variability in the presence of sulfur oxidation genes (i.e., the SOX system) which oxidize thiosulfate to sulfate and represent the potential for sulfur acquisition. We also observed variation in the pentose phosphate pathway (PPP), which is involved in the creation of aromatic amino acids, the production of nucleic acid, the generation of NADPH, interconnections with glycolysis, and C fixation. A more complete PPP may allow for species to take advantage of multiple C pathways as opposed to pathways specific to chitin breakdown, leading changes in fundamental niche size described above. Finally, numerous MSC-2 members contained variable secretion systems which, besides their understood role in virulence, export numerous proteins or other substrates outside the cell. These secretion systems are involved in the production of extracellular chitin-degrading enzymes produced by certain members of MSC-2 and are thus critical for the chitin-related interspecies interactions driving this community (30).

*Streptomyces* (Fig. 3A) contains genes for all stages of chitin metabolism (despite this species showing low growth on chitin under liquid conditions) (Fig. 1B). For example, *Streptomyces* contains chitinase genes encoding enzymes to convert chitobiose to NAG and additional genes encoding enzymes to move NAG into the central glycolysis pathway via fructose-6-phosphate. *Streptomyces* also contains NAG importers along with several other importers. N and S assimilation pathways are also present in this microorganism.

*Neorhizobium* (Fig. 3A) contains a chitin breakdown pathway, although it is not as complete as that in *Streptomyces*, and it lacks a NAG importer. Despite this, it contains a larger number of other importers and exporters as well as complete N and S assimilation pathways. *Neorhizobium* also contains a denitrification pathway as well as several genes of the SOX system.

*Dyadobacter* (Fig. 3A), a species showing growth with chitin under our conditions, contains a reduced chitin breakdown pathway that is similar to that found in *Neorhizobium* (a species for which we found little chitin growth). The main differences are the existence of GH18 (a chitinase) and GH20 (a β-hexosaminidase) in *Dyadobacter* (Fig. 3C). *Dyadobacter* also has N and S assimilation and SOX genes. This species also has a more complete PPP system than *Neorhizobium* and *Streptomyces*. However, *Dyadobacter* has very few importers and none for NAG. *Sphingopyxis* (Fig. 3A), another species showing no growth on chitin, contains a chitin breakdown pathway like *Streptomyces* along with a NAG importer (though few other importers). This species lacks a SOX system, but it does contain N and S assimilation pathways as well as a nitrate reduction set of genes. *Sphingopyxis* also contains both type I and type II sets of secretion systems.

*Ensifer* (Fig. 3B) contains perhaps the widest range of processes among the species of MSC-2 and shows good growth on chitin. Many importers are present in this *Ensifer* species, though, interestingly, not an importer for NAG. *Ensifer* contains a chitin breakdown pathway that is focused on early stages of chitin breakdown as well as N and S assimilation pathways and several genes involved in denitrification. *Ensifer* also contains more a more complete PPP system, similar to *Dyadobacter*. A type IV secretion system is also present in *Ensifer*.

*Variovorax* (Fig. 3B) has a similar genomic profile to *Ensifer*, but with an additional type II secretion system and less complete chitin decomposition and denitrification pathways. *Variovorax* has many importers and exporters, although fewer than *Sinorhizobium* (Fig. 3B), and contains a chitin breakdown pathway like *Streptomyces*, with genes critical early in the chitin breakdown process, as well as a SOX system and many uptake genes—nearly as many as *Ensifer*. *Sinorhizobium* contains an N and S assimilation pathway, but it has a much less complete SOX pathway and no denitrification genomic potential.

Finally, *Rhodococcus* (Fig. 3B) has a somewhat reduced genomic potential compared to many of the other MSC-2 isolates. The chitin breakdown genes of *Rhodococcus* are minimal, and it does not contain an enhanced PPP system as *Variovorax*, *Sinorhizobium*, *Ensifer*, and *Dyadobacter* do. *Rhodococcus* does contain N and S assimilation pathways and some nitrate reduction genes, but it has no SOX genes. *Rhodococcus* does contain both type I and type II secretion systems and some importers and exporters: more than *Dyadobacter* and *Sphingopyxis* but fewer than other MSC-2 members.

The large variation in predicted genomic potential of the MSC-2 species reflects their various phylogenies and suggests that interactions among these species may be prevalent. This may be especially true in a chitin-rich environment considering the variability in the growth of these species under conditions where chitin is the sole C source (Fig. 1B) and their genomic potential for chitin/NAG metabolism (Fig. 3C). Detailed information on genes coding for other metabolic pathways aside from chitin are shown in Fig. S1.

10.1128/msystems.00372-22.1 FIG S1 Metabolic pathways of MSC-2 constituents. MSC-2 species are shown in columns with colors matching those in Fig. 3. Metabolic pathways are shown as rows. The density of the color either indicates the number of genes encoding a particular function, as in the case of the category chitin processing, or the relative number of genes identified from a given pathway (e.g., the pentose phosphate pathway). Download FIG S1, PDF file, 0.3 MB. Copyright © 2022 McClure et al. 2022 McClure et al. https://creativecommons.org/licenses/by/4.0/ This content is distributed under the terms of the Creative Commons Attribution 4.0 International license.

### Transcriptomic analysis of MSC-2 members during community growth on chitin.

A major goal of our study was to understand how the phenotypes of species, especially decomposers such as chitin degraders, are defined by their surrounding community. To that end, we next explored how the complete MSC-2 community responded to growth on chitin as the sole C source and how a member’s role in the community shifted compared to monoculture growth. While optical density (OD) measurements were difficult to obtain due to the large amount of nonsoluble chitin used in these coculture experiments, increases in the amount of both DNA and RNA were observed across time points from 4 to 118 h (approximately a 5-day growth assay) (Fig. S2). These increases in DNA and RNA yields indicated that MSC-2 grew using chitin as the sole C source.

10.1128/msystems.00372-22.2 FIG S2 Biomass increases of MSC-2 during chitin growth. (A) DNA concentrations of MSC-2 pellets from 1 mL of growth across eight time points. Time points are shown on the *x* axis, and the DNA concentration (in nanograms per microliter) is shown on the *y* axis. Three biological replicates were examined, indicated by blue, black, and gray lines. (B) Similar analysis but showing RNA concentrations (in nanograms per microliter) across four time points. Download FIG S2, PDF file, 0.1 MB. Copyright © 2022 McClure et al. 2022 McClure et al. https://creativecommons.org/licenses/by/4.0/ This content is distributed under the terms of the Creative Commons Attribution 4.0 International license.

**FIG 4:**
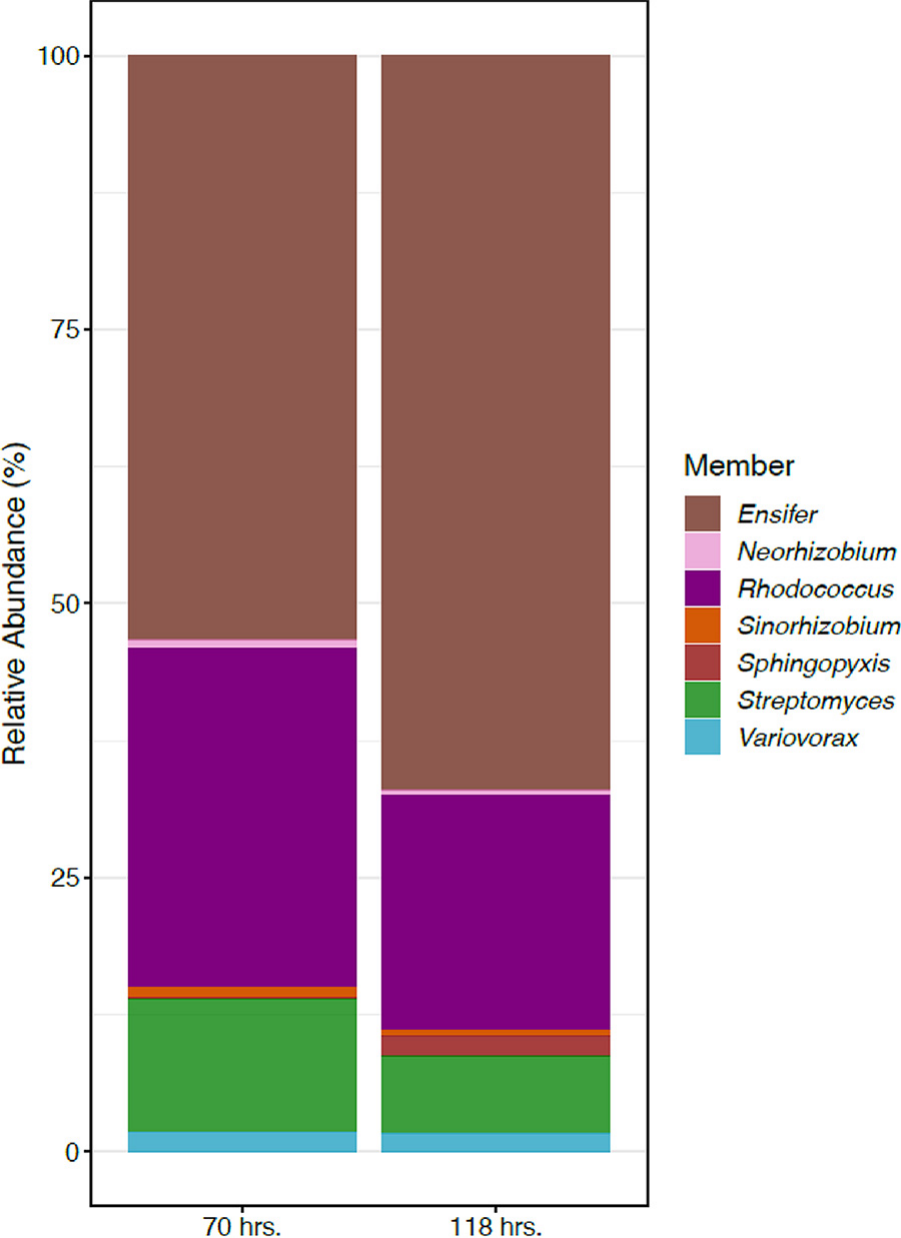
Metatranscriptomic analysis of MSC-2 community. Shown are relative abundances of RNA transcripts aligning to each of the species of MSC-2 at 70 and 118 h after the start of chitin growth.

Metatranscriptomic analysis of MSC-2 was carried out after 70 and 118 h of growth on chitin. *Ensifer* comprised the largest proportion of aligned reads at 70 h, representing 53% ± 14% of all reads (Fig. 4 and Table 2). This was followed by *Rhodococcus* (30% ± 19%), *Streptomyces* (12% ± 6%), and *Variovorax* (1.8% ± 0.66%), with the remaining species each present at less than 1% of total reads. Results at 118 h were similar to those at 70 h (Fig. 4), with the exception of *Sphingopyxis*, which increased from 0.17% ± 0.069% at 70 h to 1.61% ± 0.45% at 118 h. This 9.4-fold increase was significant with a *P* value of <0.02. Species abundance measures made using RNA alignment matched strongly with 16S amplicon abundance measures taken at 94 h. (Fig. S3). Slight differences, for example, in the case of *Rhodococcus* and *Streptomyces*, likely result from the differences in the measurement types (16S DNA versus global RNA) and not shifts in the absolute abundance of organisms at 94 h compared to 70 or 118 h. Both measures of abundance revealed that MSC-2 is a community that contains multiple species at biologically significant abundance levels. We also found that there was a strong association between species that were abundant within MSC-2 when grown together on chitin and those that had large fundamental niches. *Ensifer*, *Rhodococcus*, and *Streptomyces* were the three most abundant species and had the largest fundamental niches. (The *Rhodococcus* niche size was the same as that of *Variovorax*.) *Neorhizobium* and *Dyadobacter* were among the least abundant and had the smallest fundamental niches. This is despite the fact that *Dyadobacter* is able to grow using chitin as the sole carbon source under these growth conditions and *Streptomyces* is not.

**TABLE 2:** Metatranscriptomics of MSC-2

Species	% of abundance (RNA at 70 h)	Total no. of:		% of DEGs^*c*^
		DEGs^*a*^	Genes^*b*^	
*Streptomcyes*	12.111	48	7,143	0.67
*Neorhizobium*	0.866	128	7,622	1.68
*Dyadobacter*	0.053	0	6,281	0.00
*Sphingopyxis*	0.179	411	4,407	9.33
*Ensifer*	53.185	1937	7,069	27.40
*Variovorax*	1.793	1897	6,258	30.31
*Sinorhizobium*	0.841	895	5,833	15.34
*Rhodococcus*	30.972	3909	7,526	51.94

a Total number of differentially expressed genes (see Materials and Methods).

b Total number of genes in the genome.

c Percentage of the genome comprised of DEGs: (no. of DEGs/no. of genes in genome) × 100.

10.1128/msystems.00372-22.3 FIG S3 16S analysis of MSC-2 during chitin growth. Shown are relative abundance levels of MSC-2 species based on 16S rRNA sequencing of DNA 94 h after the start of chitin growth. Download FIG S3, PDF file, 0.03 MB. Copyright © 2022 McClure et al. 2022 McClure et al. https://creativecommons.org/licenses/by/4.0/ This content is distributed under the terms of the Creative Commons Attribution 4.0 International license.

To determine which species were most transcriptionally active, differentially expressed genes (DEGs) were identified for each species of MSC-2 (Table 2 and Fig. S4). This analysis revealed that *Rhodococcus* had the highest degree of differential gene expression (as measured by the percentage of DEGs in the genome, 51%). This was followed by *Variovorax* (~30%), *Ensifer* (~27%), and *Sinorhizobium* (~15%). *Streptomyces* and *Neorhizobium* showed the smallest changes in DEGs. This indicates that species with low relative abundances in MSC-2 (e.g., *Variovorax* at ~2% and *Sinorhizobium* at ~0.7%) could still contain a large number of DEGs and potentially contribute to community-level processes. Conversely, abundant species such as *Streptomyces* were shown to have minimal differential gene expression, suggesting that while they were highly abundant, they may contribute only a small number of processes to the system during growth on chitin at the time points measured. This apparent lack of contribution may be specific to the later stages of chitin growth examined here; their contributions may be more important early in the growth timeline when direct breakdown of chitin is more critical to providing C to the system.

10.1128/msystems.00372-22.4 FIG S4 Volcano plots of DEGs in MSC-2. Volcano plots for each species are shown. Green dots indicate DEGs (defined as those genes showing a greater than 2-fold change in expression with an adjusted *P* value of <0.05). Blue dots indicate genes with an adjusted *P* value of <0.05 but a fold change value of <2. Black dots indicate genes with an adjusted *P* value of >0.05 and a fold change value of <2. *Dyadobacter* had no DEGs and is not shown. Download FIG S4, PDF file, 0.1 MB. Copyright © 2022 McClure et al. 2022 McClure et al. https://creativecommons.org/licenses/by/4.0/ This content is distributed under the terms of the Creative Commons Attribution 4.0 International license.

**FIG 5:**
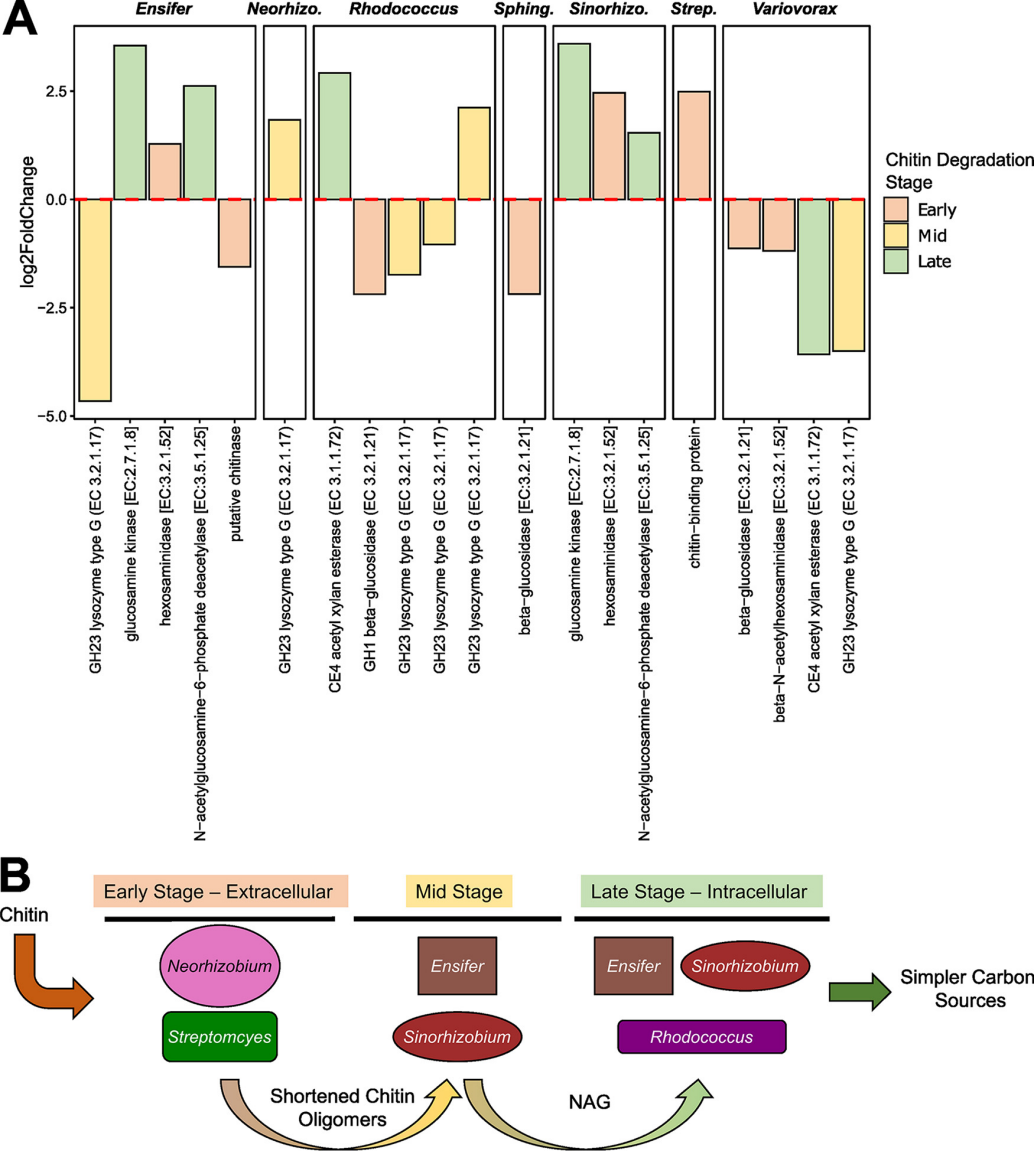
Chitin metabolism-related metatranscriptomic data of MSC-2 during chitin growth. (A) Increased or decreased expression of genes in chitin-related pathways is shown for each species comparing the 70-h and 118-h incubations with chitin. Positive log_2_ fold changes indicate that the gene is more highly expressed at 118 h versus 70 h. Only genes showing statistically significant changes in expression are included. Genes are colored by the chitin breakdown stage they have been assigned to, where “early” refers to enzymes degrading larger molecules and “mid” and “late” refer to downstream enzymes or assimilation pathways. (B) Schematic depicting the hypothesized route of chitin decomposition through MSC-2 based on metatranscriptomic data.

We next examined which functional processes related to chitin metabolism were expressed by each of the species in MSC-2 (Fig. 5A), by comparing 70 h to 118 h of growth on chitin. Growth at this later time point caused an upregulation of chitin binding genes in *Streptomyces*, *Neorhizobium*, and to a more mixed degree, *Rhodococcus*, and an upregulation of genes encoding enzymes that are involved in conversion of shorter chitin or chitobiose polymers to NAG monomers in *Sinorhizobium* and *Ensifer*. At the same time, genes involved with the processing of NAG showed an increase in expression in *Sinorhizobium*, *Rhodococcus*, and *Ensifer* (Fig. 5A). Overall, when DEGs of chitinase decomposition pathways were compared over time (70 h compared to 118 h), *Streptomyces* and *Neorhizobium* contributed to carrying out processes early in the metabolism of chitin (expressing proteins that bind chitin directly or expressing known chitinases), *Sinorhizobium* and *Ensifer* contributed processing midway between chitin breakdown and NAG metabolism, and *Sinorhizobium*, *Rhodococcus*, and *Ensifer* contributed to later stages of conversion of NAG to even simpler C and N sources (e.g., NAG-6-P to glucosamine-6-P which leads to fructose-6-P and the glycolysis/gluconeogenesis pathway) (Fig. 5B). Transcriptomic expression of other pathways for MSC-2 members is shown in Fig. S5. These observations address the hypothesis that species in a chitin-degrading community tend to organize themselves into specific roles. Not all species express all chitin breakdown pathways, even if they possess them: rather, certain species express only certain pathways, likely to maximize both community and constituent growth.

10.1128/msystems.00372-22.5 FIG S5 Functional annotation of metatranscriptomic data of MSC-2. A box and whisker plot is shown for each species, with each box assigned to a particular functional category. The red line indicates no fold change. Box areas or mean lines above this red line indicate increased expression of genes at 118 h compared to 70 h. *Dyadobacter* had no DEGs. Download FIG S5, PDF file, 0.1 MB. Copyright © 2022 McClure et al. 2022 McClure et al. https://creativecommons.org/licenses/by/4.0/ This content is distributed under the terms of the Creative Commons Attribution 4.0 International license.

Overall, these analyses revealed that a species abundance is tightly linked to fundamental niche size, that multiple species may be transcriptionally active, despite low abundances, and that certain other species with high abundances can have low transcriptional activities. The most active species in MSC-2 were *Rhodococcus*, *Variovorax*, and *Ensifer*, and the major species likely contributing to initial, energy-intensive processes in chitin degradation were *Neorhizobium* and *Streptomyces* (Fig. 5). Despite the specific regulation of chitin processes in *Streptomyces*, other pathways in this organism showed very little transcriptional regulation at the time points sampled. It is also of note that we did not find *Streptomyces* to be able to grow on chitin in monoculture under the conditions of our experiment. Interactions with other species of MSC-2 in a community may thus contribute to growth of Streptomyces in coculture compared to axenically. This observation helps to answer another of our major hypotheses: that the phenotype of species (i.e., whether it acts as a degrader of chitin and can grow using chitin as the sole carbon source) is strongly influenced by surrounding community members. The chitin growth phenotype is not expressed by *Streptomyces* in monoculture but is expressed in coculture with other MSC-2 species.

### Metabolomic analysis of MSC-2 under chitin conditions.

**FIG 6:**
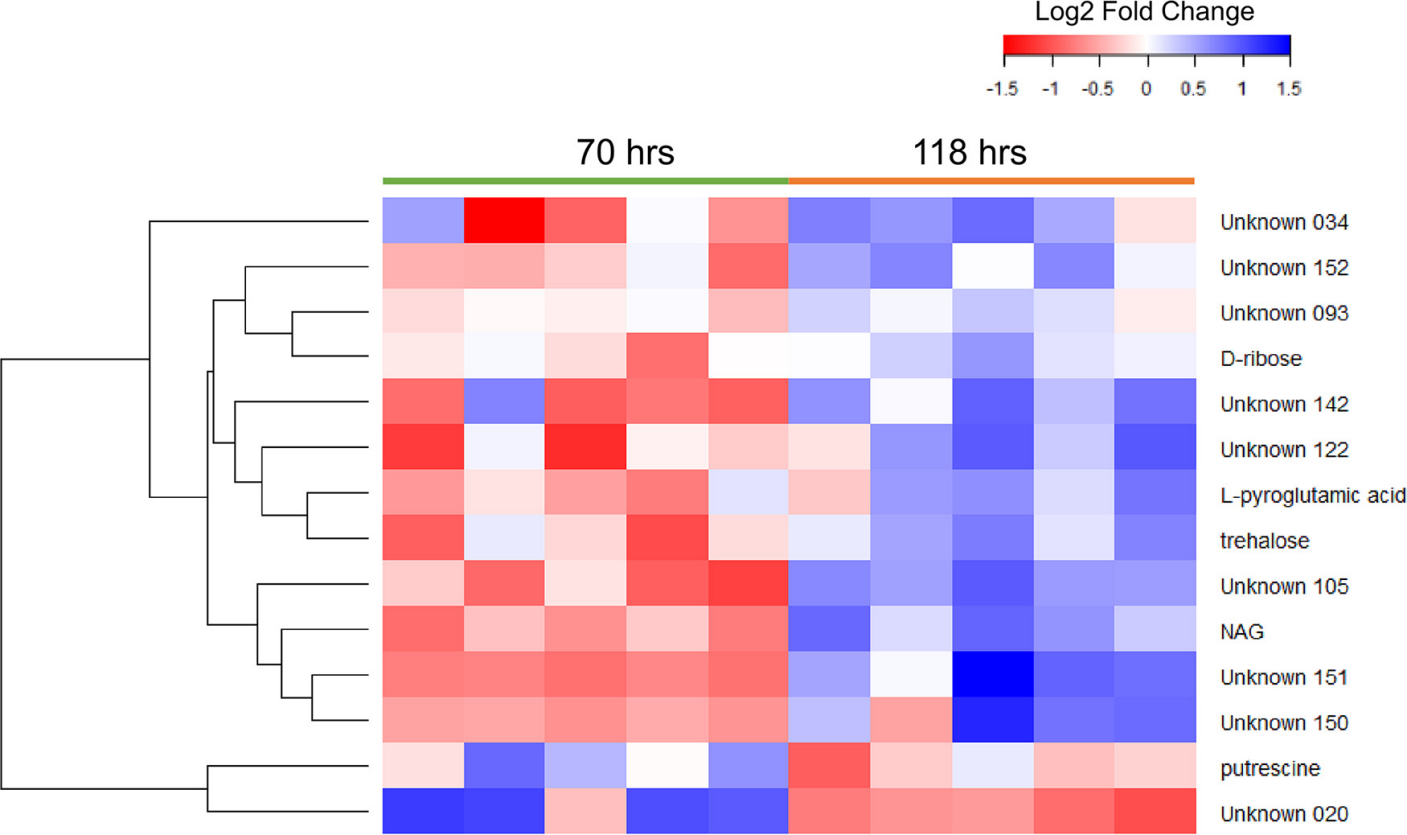
Metabolite profile of MSC-2 in chitin. The heat map shows metabolite abundances at 70 h (leftmost 5 columns) and 118 h (rightmost 5 columns). Blue indicates an increase from the mean of all 10 samples (five at 70 h and five at 118 h); red indicates a decrease from the mean. The color key at the top shows fold changes associated with each color. The dendrogram on the left side shows clustering of metabolites by similarity of response.

To gain a better understanding of downstream products of chitin breakdown in this community intracellular metabolomic analysis was carried out at the same time points as the metatranscriptomic analysis (70 and 118 h after chitin addition). Metabolic profiles of the communities shifted over time (Fig. S6 and Tables S3 and S4). In total, 14 metabolites were significantly different between the two time points, with most showing increased abundances at 118 h compared to 70 h (Fig. 6). Trehalose, l-pyroglutamic acid, d-ribose, and NAG were among the identified metabolites showing increased abundances. Several other metabolites also showed increased abundance, but their identity could not be confirmed due to the presence of only a mass spectral match and not an accompanying retention time or index match. However, provisional annotation of these unknowns was possible, and they included *N*-acetyl-d-galactosaminitol (unknown 105), *N*-acetyl-d-galactosamine (unknown 151), and a disaccharide (unknown 122). NAG, and related annotated molecules, were of particular interest as they are some of the main breakdown products of chitin, and in fact NAG and *N*-acetyl-d-galactosaminitol showed the largest fold changes (6.55- and 6.64-fold higher at 118 h versus 70 h, respectively). Among metabolites showing decreased abundances at 118 h were an unknown metabolite (likely hydroxylamine) and putrescine. Decreases in putrescine may be related to this molecule’s link to chitin breakdown (31). At higher chitin levels (70 h), chitin is being degraded and putrescine levels are high. Later in the growth curve (118 h), chitin levels are lower, chitin breakdown slows, and putrescine levels fall. Fold changes for all significantly shifted metabolites are shown in Table S3. Raw data for all detected metabolites (both known and unknown) are shown in Table S4.

10.1128/msystems.00372-22.6 FIG S6 PCA (principal-component analysis) plot of metabolic abundance data in MSC-2. Green dots indicate samples collected at 70 h of growth in chitin. Red dots indicate samples collected at 118 h of growth in chitin. Download FIG S6, PDF file, 0.03 MB. Copyright © 2022 McClure et al. 2022 McClure et al. https://creativecommons.org/licenses/by/4.0/ This content is distributed under the terms of the Creative Commons Attribution 4.0 International license.

10.1128/msystems.00372-22.9 TABLE S3 List of metabolites showing statistically significant fold change at 70 h versus 118 h. Download Table S3, PDF file, 0.1 MB. Copyright © 2022 McClure et al. 2022 McClure et al. https://creativecommons.org/licenses/by/4.0/ This content is distributed under the terms of the Creative Commons Attribution 4.0 International license.

10.1128/msystems.00372-22.10 TABLE S4 List of all detected metabolites and their abundance in all samples. Download Table S4, PDF file, 0.4 MB. Copyright © 2022 McClure et al. 2022 McClure et al. https://creativecommons.org/licenses/by/4.0/ This content is distributed under the terms of the Creative Commons Attribution 4.0 International license.

### Integration of metatranscriptomic and metabolomic data to infer community processes.

**FIG 7:**
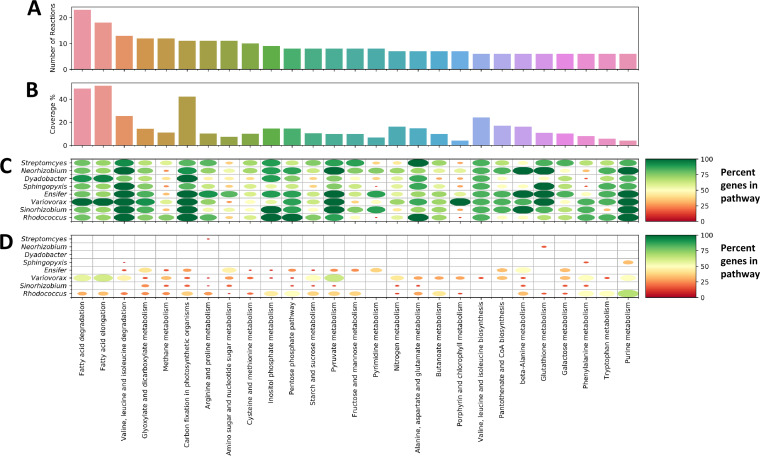
Integrated metatranscriptomic and metabolomic analyses of MSC-2. (A) Number of reactions (*y* axis) predicted using MEMPIS with metabolomic and metatranscriptomic data in each functional category (*x* axis). (B) The coverage of all of the reactions in the functional category is shown as a percentage (e.g., fatty acid degradation has ~20 reactions predicted within this functional category, and this number accounts for ~45% of the total known reactions in this functional category). (C) Genomic potential of each species of MSC-2 in each functional category. Larger, darker green circles indicate that a greater percentage of the genes within the indicated functional category are found in the genome of the MSC-2 species. (D) Expressed genes (determined from metatranscriptomic data) from each species in MSC-2 in each functional category. Larger, darker green circles indicate that a greater percentage of the genes within the indicated functional category are expressed by the MSC-2 species.

The collection of multiple omics data sets during growth of MSC-2 on chitin allowed us to take an integrated approach for downstream analysis using Metabolite-Expression-Metabolic Network Integration for Pathway Identification and Selection (MEMPIS), a tool that combines metabolic and metatranscriptomic data to determine which functions a community is expressing (32, 33) (Fig. 7A and B). The integration of metatranscriptomic data and metabolomic data also provided a more robust way to identify expressed processes. Previous work has pointed out the sometimes spurious link between expressed genes and the functions they encode (34); our incorporation of metabolomic data will help alleviate that issue and improve accuracy. This analysis revealed that fatty acid metabolism and amino acid metabolism had the most coverage, indicating that a wide range of processes in these categories had the potential to be activated in the MSC-2 system. Regarding processes likely related to chitin breakdown, the amino sugar metabolism pathway also had many expressed metabolites and genes, but the coverage was small, suggesting that only certain parts of this broad pathway were activated. In contrast, N metabolism had few genes and metabolites being expressed, but the overall coverage of this pathway was relatively high, showing that much of the known pathway was being expressed.

The metatranscriptome was then screened to determine which species were expressing which genes (Fig. 7C and D). *Variovorax* expressed almost all the detected genes involved in fatty acid metabolism, with smaller contributions from *Rhodococcus*. *Variovorax* was also the major driver of pyruvate metabolism. *Rhodococcus* carried out purine metabolism, tryptophan metabolism, and phosphate processes. *Ensifer* participated in fewer processes (glyoxylate, pyrimidine, and alanine metabolism) than *Variovorax* and *Rhodococcus*, despite *Ensifer* being the major constituent of MSC-2 when measured in both DNA and RNA data sets. Looking specifically at processes centering on chitin and NAG metabolism, *Variovorax* was the main contributor to N metabolism and *Ensifer* to amino sugar metabolism.

In some cases, MSC-2 species had outsized roles in certain processes compared to their genomic potential for such processes (Fig. 7C and D). The large role of *Ensifer* in glyoxylate metabolism contrasts with the fact that *Variovorax* had much more genomic potential to participate in these processes but did not in this experiment, at least as measured at the transcriptional level. Similarly, *Variovorax* had the greatest gene expression related to starch metabolism, despite having less genomic potential for that process than *Rhodococcus* and *Streptomyces*. Likewise, *Rhodococcus*’ expression of genes associated with alanine metabolism occurred despite it having less genomic potential for that process than *Neorhizobium*, *Ensifer*, *Variovorax*, and *Sinorhizobium*. All of this suggests that species occupy unique niches within MSC-2 based not on each member’s genomic potential but on their expression of specific gene sets designed to maximize their growth and/or survival as they interact with other species either cooperatively or competitively.

## DISCUSSION

The native soil microbiome is a complex community consisting of thousands of species with potentially millions of interactions between them. Because no species exists in isolation, it is these interactions that lead to the emergent properties of the soil microbiome and its role in cycling C and N substrates. Here, we sought to better understand organismal phenotypes, the roles of individual species, and how this is affected by fellow community members. By focusing on a well-defined, simplified consortium (MSC-2) that was derived from a naturally evolved soil consortium (MSC-1) (26), we were able to investigate species-specific contributions to community metabolism, using the decomposition of chitin as our study system.

One of our major findings is that within MSC-2, chitin degradation potential (as measured by the ability to grow with chitin as the sole C source in monoculture) is not the sole or even the major determinant of how abundant (as measured by metatranscriptomic analysis and evaluating how many reads align to a given species) a member will be in the community when grown on a complex carbon source like chitin. Analysis of the fundamental and realized niches of MSC-2 members as well as the growth advantages and disadvantages of member species in a community versus axenic growth showed that ranking the species from largest to smallest fundamental niche (Fig. 2C) aligned very well with ranking by the abundances of each species (Fig. 4 and see Fig. S3 in the supplemental material). For some species, we noticed a lack of abundance in MSC-2, even though the ability of these species to grow on chitin had been shown. The trade-off between the ability to grow on chitin in monoculture and abundance in the MSC-2 community is especially striking for *Sinorhizobium*. Although this species can grow on chitin and expresses chitin binding genes, its relative abundance in MSC-2 was low. This contrast between chitin growth ability and community abundance (during growth on chitin) may be because the fundamental niche of *Sinorhizobium* is small compared to those of other species such as *Ensifer.* As a result of *Ensifer*’s larger niche, while it may not drive chitin degradation, it may be able to grow on breakdown products provided by initial chitin degraders such as *Sinorhizobium*. In contrast, *Sinorhizobium*, with its smaller fundamental niche, is not able to take great advantage of the range of chitin breakdown products and is limited to focusing on chitin itself, which is constantly falling in concentration as the experiment proceeds and is difficult to metabolize, all of which may lead to less growth of *Sinorhizobium*.

The observation that microbes with large fundamental niches may survive off chitin breakdown products opens a new question: what do these secondary consumers provide to the community in exchange for receiving breakdown products? Several possibilities emerge. First, secondary consumers may survive off C and N sources that degraders are generating but cannot metabolize themselves as has been seen with phenylalanine and Escherichia coli (35, 36). Alternatively, consumption of metabolites by secondary consumers may relieve stoichiometric pressure on critical enzymatic reactions of degraders, allowing for more continued degradation of chitin (22). This may especially be the case for *Streptomyces* as high levels of NAG have been shown to repress chitin breakdown in this organism (17). Another possibility is that secondary consumers may produce important vitamin cofactors such as cobalamin. While this vitamin is critical to the soil microbiome, only a small number of bacteria and archaea produce B_12_ (23), suggesting that these B_12_ producers are important to community growth even if they do not provide C or N. Our experiments here included excess B_12_, but this may be the case for other vitamins or secondary metabolites that are limiting. For example, the major chitin degraders *Neorhizobium* and *Streptomyces* have importers for both branched-chain amino acids and xylose molecules (Fig. 3). Gaining these from nondegraders would be energetically favorable for these species. It is important to note that these fundamental niche conclusions are possible only after defining this soil-derived consortium and building metabolic models for each individual species. Such a detailed understanding of a soil microbial consortium will help to answer several questions beyond what we show here and will allow us to begin to bridge the gap between the molecular details possible with laboratory experiments and the more complex, but translational, results from direct field analysis.

Our investigations of MSC-2 provide a deeper understanding of how species that cannot grow on complex organic carbon axenically may alter their phenotypes and contribute to decomposition in the context of other community members. For example, under the growth conditions of this experiment *Streptomyces* was not able to grow in monoculture on chitin as the sole C source. This result was consistent for each of the three *Streptomyces* strains that we isolated (Fig. 1B). This lack of growth in monoculture combined with the observed high abundance of *Streptomyces* within MSC-2 and our metatranscriptomic analysis suggests that *Streptomyces* contributes directly toward chitin decomposition in MSC-2, but the energy limitations presented by monoculture growth on chitin prohibit enzyme production. Moving a species from an environment with labile C sources (our initial growth on R2A medium) to an environment with chitin as the only C substrate may not allow *Streptomyces*, when grown in monoculture, to generate chitin-degrading enzymes. *Streptomyces* may then be “stuck” with abundant chitin but not enough nutrients to allow for the initial synthesis of chitinases. We designed the experiment to account for this possibility by including a 3-day period of growth in chitin and NAG to allow for species to acclimate from richer medium (R2A) to chitin-only minimal medium. While this may have been useful for some organisms, it may have had the opposite effect on *Streptomyces* as chitinase production in *Streptomyces* species has been shown to be repressed by the presence of NAG (17). However, in contrast to monoculture, in the context of the complete MSC-2 community, other species can initially degrade chitin, provide breakdown products, allow *Streptomyces* to grow and make its own chitin-degrading enzymes, and contribute to chitin degradation later in the experimental timeline.

These conclusions emphasize the differences between inherent phenotypes of microorganisms in isolation (e.g., no growth of *Streptomyces* on chitin in monoculture) versus the phenotypes expressed when grown as a community (e.g., growth of *Streptomyces* on chitin in a community). There is an extension of this observation that is more closely related to soil ecology: that phenotypes of a species are dependent on the nature of the surrounding community. As communities are constantly shifting in soil as a function of environmental changes, testing this hypothesis further is critical for a deeper understanding of the community interactions that are at the foundation of soil microbial ecology. Emergent properties are often thought of as functions emerging from the community as a whole (37). However, species within a community also show phenotypic properties that emerge only when they are grown with certain other species. In fact, emergent properties of individual species are likely what leads directly to emergent properties of the community as a whole. These results also emphasize the importance of examining species in both the context of other microorganisms and axenically (where possible) to fully understand their contributions to community metabolism and growth. Incorporation of species’ actions during monoculture versus community growth is also critical for applying what is learned with model communities back to the field, where no species exist in isolation and community growth is the rule. While our development of MSC-2 will help bridge the gap between laboratory and field, it should be noted that this is a community of relatively few isolates that are combined from individual strains, so the discoveries learned here may not represent the phenotypes expressed in the field. However, two aspects of our studies suggest that MSC-2 is a valuable tool for understanding soil microbial ecology. First, while MSC-2 is comprised of isolates, these isolates were drawn from a naturally evolved community derived from the complete soil microbiome (MSC-1) (26). Members of MSC-1 (and therefore MSC-2) were chosen not by us by rather by existing interactions of soil microbiology that drove community assembly. Therefore, constituents of MSC-2 are likely to participate in interactions that are, in part, representative of processes of the native soil microbiome. Second, our use of MEMPIS in these studies allows us to apply the same methods and the same omics measurements between the lab (MSC-2) and the field (native soil microbiome). This will allow us to map processes happening in both systems and highlight where MSC-2 can be used to better understand and interpret native soil microbiome processes and where it cannot.

In summary, we describe a model chitin-degrading consortium and use multiomics analysis as well as axenic versus coculture studies to delineate the role of individual species within this consortium. Chitin-degrading members likely supported non-chitin degraders through sharing of chitin breakdown products. Because of this sharing, the main driver of a species growth and success was not the ability to carry out specific metabolic processes related to abundant C sources (chitin breakdown), but rather which species could take the most advantage of shared breakdown products. Also related to community growth, we show that the phenotype of a species, regarding its role as a primary decomposer or not, is driven by the surrounding community. The data and conclusions gathered here will be instrumental in guiding future experiments focused on combining individual constituents of MSC-2 in pairwise combinations and performing experiments in which members of MSC-2 are left out. These conclusions will also be critical to our understanding of how native soil microbiomes process soil organic C, especially substrates such as chitin that drive interactions and metabolite sharing, and how these processes may shift as community membership changes as a function of both biotic and abiotic pressures. Application of these conclusions to the native soil microbiome will greatly expand our ability to identify what the keystone species are, which may have the greatest advantage for growth, and how these communities are organized to promote C cycling in natural settings.

## MATERIALS AND METHODS

### Isolation and analysis of new constituents of MSC-2.

These experiments build on a previously described field-derived, reproducible consortium, MSC-1. MSC-1 is a chitin-enriched consortium containing ~35 species for which we have identified keystone species and a preliminary interaction network. To test how individual members of MSC-1 are contributing to community metabolism and growth, we developed a more-reduced-complexity consortium, MSC-2, as a subset of MSC-1. Isolates of MSC-1 for use in MSC-2 were collected as described previously (26) through plating on R2A agar medium followed by selection of colonies with morphological differences and 16S rRNA amplicon sequencing to determine identity down to the genus level. For genome assembly of isolates, DNA was extracted using the ZymoBIOMICS DNA microprep kit (Zymo, Irvine, CA). DNA was then sequenced using Illumina technology, collecting ~630 Mbp per genome (see Table S1 in the supplemental material). Reads were quality trimmed using bbduk (part of the bbtools package (https://sourceforge.net/projects/bbmap/) and the following parameters: int ow k = 27 hdist = 1 qtrim=f ref=sequencing_artifacts.fa.gz, phix174_ill.ref.fa.gz minlen = 35. The SPAdes assembler (v.3.13.1) (38) was then used to assemble reads with the following parameters: -k 21,33,55,77,99,127 –careful. Assembled genomes were analyzed using the DRAM (Distilled and Refined Annotation of Metabolism) pipeline in the reduced memory mode (–skip_uniref) (39). As part of the DRAM pipeline, KEGG Orthology (KO) numbers were assigned using the KOfam database, which were subsequently used to visualize pathways using the KEGG mapper tool (40, 41). The DRAM pipeline additionally provides broad metabolic categories for each annotated gene. Using R and the associated ggplot2 R package (https://ggplot2.tidyverse.org/), these categories were parsed across each genome and a heat map of functional potential was generated. Taxonomy was assigned using the standard classification workflow (classify_wf) of the Genome Taxonomy Database toolkit (GTDBtk) v.1.4.1, and GTDB reference data v.r95 (42). Because the final species-level taxonomy may shift as the GTDB reference data are updated, the members of MSC-2 are referred to here primarily by their genera. The eight isolates comprising MSC-2 are shown in Table 1, were chosen to maximize genomic diversity, and include both chitin degraders and nondegraders.

### Cultivation of MSC-2 species individually under variable C and N sources.

Each MSC-2 strain was cultivated on R2A agar plates for 3 days prior to starting a growth assay for a particular C source. Multiple C sources were tested to generate fundamental niche profiles for each species. The resulting colonies on R2A were collected and washed three times in M9 (containing no C source and 18 mM NH_4_Cl) with centrifugation (5,000 rpm for 5 min) between each wash. Each strain was then resuspended in M9 with 18 mM NH_4_Cl and the C source under analysis to a final OD_600_ of 0.1. For growth with chitin experiments, colloidal chitin from shrimp shells (Megazyme, Bray, Ireland; product code P-CHITN) was used as the sole C source at a final concentration of 100 ppm (0.1 mg/mL). This amount was chosen so that insoluble chitin interfered as little as possible with OD readings. For all other C sources (glucose, C_6_H_12_O_6_; NAG, C_8_H_15_NO_6_; serine, C_3_H_7_NO_3_; glycine, C_2_H_5_NO_2_; alanine, C_3_H_7_NO_2_; maltose, C_12_H_22_O_11_; xylose, C_5_H_10_O_5_; glutamate, C_5_H_9_NO_4_; sucrose, C_12_H_22_O_11_; fructose, C_6_H_12_O_6_; and arabinose, C_5_H_10_O_5_), the final concentration used was 10 mM. We standardized by molarity of substrates, rather than the mass of C, because our primary goal was comparing growth among species on the same C source, not necessarily growth among C sources for each species; the latter was addressed via metabolic modeling (described below). Following inoculation with the C source, each strain was grown in biological triplicates in a plate reader with OD measurements collected every 20 min for 5 days. Each experiment contained a no-carbon control, with the OD of these wells being used to correct OD values in growth wells. Plates were shaken at 180 rpm during growth, and cultivation was carried out at 20°C.

### Metabolic modeling of MSC-2 species.

We developed metabolic models of the eight isolates using the Department of Energy Systems Biology Knowledgebase (KBase) modeling pipeline (27). We first constructed drafted models using the “Build Metabolic Model” app, which were iteratively gapfilled using the “Gapfill Metabolic Model” app to match predicted biomass production with experimentally observed growth phenotypes in M9 minimal media. The resulting isolate models were highly consistent with experimental data, correctly predicting 95% of growth and nongrowth observations. With these metabolic models, we performed flux balance analysis (FBA) (43) to predict biomass yields (grams dry weight per millimole of C source) from various carbon sources.

A mixed-bag metabolic network was constructed by merging the eight isolate models without accounting for species boundaries (44). Using comparative growth simulations with isolates and mixed-bag models, we determined fundamental and realized niches of MSC-2 species. To do so, we first identified C sources that are included in the uptake reactions of the mixed-bag model. For each of those C sources, we subsequently performed FBA using isolate and mixed-bag models to determine whether individual species and the community utilize them for growth. All C sources that support biomass production in individual isolate models were recorded as part of an isolate’s fundamental niche; C sources that the mixed-bag model can utilize for growth were recorded as part of the realized niche, which is common for all isolates.

### Cultivation of MSC-2 community with chitin.

Each MSC-2 strain was cultivated on R2A agar plates for 3 days. Each strain was resuspended in M9 liquid medium (all M9 media in these experiments contained 18 mM NH_4_Cl) containing 100 ppm chitin and 10 mM NAG to a final OD of 0.1. After 3 days of growth, cells were collected by centrifugation and washed three times in M9 with centrifugation between washes. Strains were then resuspended to a final OD of 1.0 in M9. Three hundred fifty microliters of each strain was added to a single 100-mL flask containing 30 mL of M9 and 500 ppm (0.5 mg/mL) chitin. Five biological replicate flasks, labeled A to E, were started and incubated at 20°C for 5 days. A larger amount of chitin was used for coculture growth as optical density data did not need to be gathered.

For DNA samples, three biological replicates (from flasks A to C) of 1-mL samples were collected at 0, 8, 16, 34, 46, 70, 94, and 118 h after the start of the experiment. Sample time points were chosen based on visual confirmation of growth of MSC-2 community and growth kinetics of MSC-2 constituents (Fig. 1B). Samples were centrifuged as described above, the supernatant was discarded, and the pellet was flash frozen in liquid N_2_ and stored at −80°C. For RNA, three biological replicates (from flasks A to C) of 2-mL samples were collected at 0, 34, 70, and 118 h after the start of the experiment. Samples were stored as for the DNA samples. For metabolites, five biological replicates (from flasks A to E) were collected at 70 and 118 h after the start of the experiment. Samples were stored as for the DNA samples. These later time points were chosen for omics analysis as we predicted that chitin breakdown would need several days to reach full efficiency and because biomass levels were higher at these later time points, which led to better metatranscriptomic and metabolic analyses. DNA was extracted using the ZymoBIOMICS DNA microprep kit. RNA was extracted using the ZymoBIOMICS RNA microprep kit with on-column DNase treatment. Metabolites from the cell pellets were extracted using the MPLEx extraction protocol as described previously (45).

### Multiomic analysis of MSC-2 during growth on chitin.

Metatranscriptomic analysis was carried out on the three replicates of extracted RNA at 70 and 118 h using Illumina technology and paired-end reads of 300 bp in length. This number of replicates was chosen to maximize our statistical analysis within the resources available for these experiments. As there were differences in the growth rates of MSC-2 species and certain members did not reach their highest densities until up to 109 h after the start of the experiment (based on our monoculture growth data) (Table 1), these time points were chosen so that all MSC-2 members would have a chance to grow to optimal levels prior to multiomic analysis. Total reads and alignment values from each sample are shown in Table S2. Reads were aligned to a concatenated metagenome of each of the eight species individual genomes using the Burrows Wheeler Aligner (46). The resulting SAM files were then converted to raw count files using HTSeq (47). Transcripts were normalized using the DESeq2 package within the statistics program R (48), which was also used to identify differentially expressed genes (DEGs), which were defined as genes with an adjusted *P* value of ≤0.05 and absolute fold change value of >2 comparing 70 h to 118 h. Normalization was applied to each species separately so that abundance changes would not be misinterpreted as gene expression changes. We also analyzed 16S rRNA gene amplicons on two replicates of extracted DNA at 94 h using Illumina technology and proprietary primers that cover the V3 and V4 hypervariable regions with a total read length of 500 bp. A single replicate was used here since we sought mainly to gain a confirmation of abundance levels as determined by our metatranscriptomic levels above. This was also why we chose a midpoint of 94 h between our metatranscriptomic time points of 70 and 118 h. Total reads from each sample are shown in Table S2. 16S rRNA amplicon sequences were analyzed using QIIME2 (v.2021.4) (49), and the SILVA database (v.138) (50) was used during taxonomy assignment (*q2-feature-classifer*).

10.1128/msystems.00372-22.8 TABLE S2 Alignment results of 16S and metatranscriptomic analyses. Total sequenced DNA and RNA reads are indicated as well as how many RNA reads aligned to the genomes of each species of MSC-2 and the alignment percentage. Download Table S2, PDF file, 0.1 MB. Copyright © 2022 McClure et al. 2022 McClure et al. https://creativecommons.org/licenses/by/4.0/ This content is distributed under the terms of the Creative Commons Attribution 4.0 International license.

Metabolomic analysis of MSC-2 was carried out by gas chromatography-mass spectrometry (GC-MS) analysis as described previously (51), with the samples blocked and analyzed in random order for each experiment. GC-MS raw data file processing was done using Metabolite Detector software, and metabolites were identified by matching experimental spectra and retention indices to an augmented version of FiehnLib (52). The NIST 14 GC–MS library was also used to cross-validate the spectral matching scores obtained using the Agilent library and to provide identifications of unmatched metabolites. As metabolomic analysis is sometimes imperfect due to noise in the signal (53), we collected five biological replicates for these experiments to increase our statistical power and confidence.

Metabolite-Expression-Metabolic Network Integration for Pathway Identification and Selection (MEMPIS) (32) was used to identify specific reaction pathways and associated taxon-specific genes during chitin decomposition. We integrated transcriptomic and metabolomic data measured from MSC-2 using MEMPIS with the mixed-bag metabolic model reconstructed in KBase. The MEMPIS algorithm was implemented in Python using Pyomo (54), a Python-based open-source software package to model optimization problems and pass the problem to solvers on the NEOS server (55). COBRApy (56) was used to read a systems biology markup language (SBML) file for loading the mixed-bag model and to determine the reactions associated with these overexpressed genes using gene-protein-reaction (GPR) rules defined in the mixed-bag model. ModelSEED identifiers were used to map metabolites and these reactions to the mixed-bag model. We used CPLEX v20.1.0.0 on the NEOS server to solve the minimization problem in MEMPIS.

### Data availability.

All sequencing data has been uploaded onto DataHub. Genome accession numbers for MSC-2 species in GenBank are uploaded at: https://data.pnnl.gov/group/nodes/dataset/33234. 16s amplicon data is uploaded at: https://data.pnnl.gov/group/nodes/dataset/33231. Metatranscriptomic data is uploaded at: https://data.pnnl.gov/group/nodes/dataset/33232.
