# Supplementary material for: Interaction Networks Are Driven by Community-Responsive Phenotypes in a Chitin-Degrading Consortium of Soil Microbes
Source: mSystems. 2022 Sep 26;7(5):e00372-22. doi: 10.1128/msystems.00372-22 (PMC9599572; doi:10.1128/msystems.00372-22)
Supplement: TABLE S1 [file msystems.00372-22-s0007.pdf]

Supplementary Table 1. List of Species isolated from MSC-1

Strain Designation	Taxonomy	Total Reads for Genome Assembly	Total Mbp for Genome Assembly
PNNL_MSC-1_Str_001	*Streptomyces*	1460154	730
PNNL_MSC-1_Str_003	*Ensifer*	1612474	806
PNNL_MSC-1_Str_005	*Neorhizobium*	1187525	594
PNNL_MSC-1_Str_007	*Dyadobacter*	1469912	735
PNNL_MSC-1_Str_008	*Sphingopyxis*	1289506	645
PNNL_MSC-1_Str_011	*Ensifer* (Unique Strain 2)	1200701	589
PNNL_MSC-1_Str_012	*Variovorax*	1198753	585
PNNL_MSC-1_Str_014	*Sinorhizobium*	1189235	586
PNNL_MSC-1_Str_015	*Dyadobacter* (Unique Strain 2)	1186754	582
PNNL_MSC-1_Str_016	*Rhodococcus*	1194339	577
PNNL_MSC-1_Str_017	*Streptomyces* (Unique Strain 2)	1176343	581
PNNL_MSC-1_Str_018	*Streptomyces* (Unique Strain 3)	1215266	590
