# Supplementary figures and images for: Interaction Networks Are Driven by Community-Responsive Phenotypes in a Chitin-Degrading Consortium of Soil Microbes
Source: mSystems. 2022 Sep 26;7(5):e00372-22. doi: 10.1128/msystems.00372-22 (PMC9599572; doi:10.1128/msystems.00372-22)

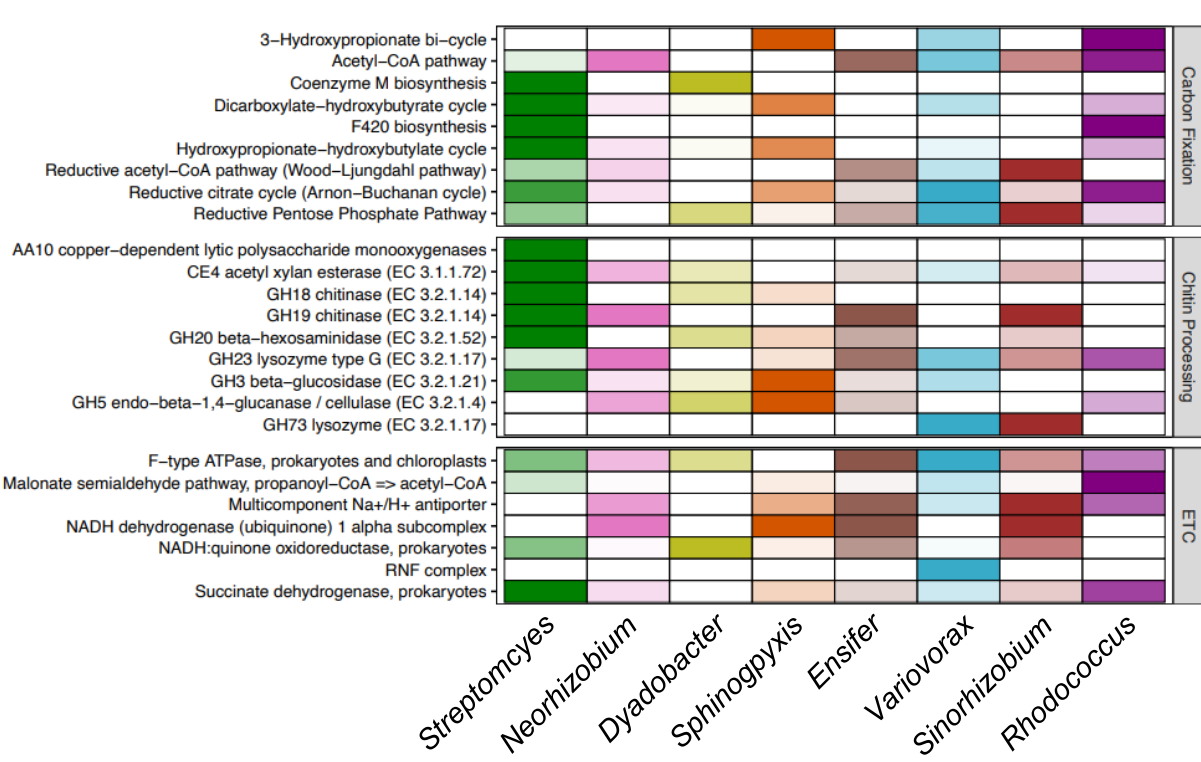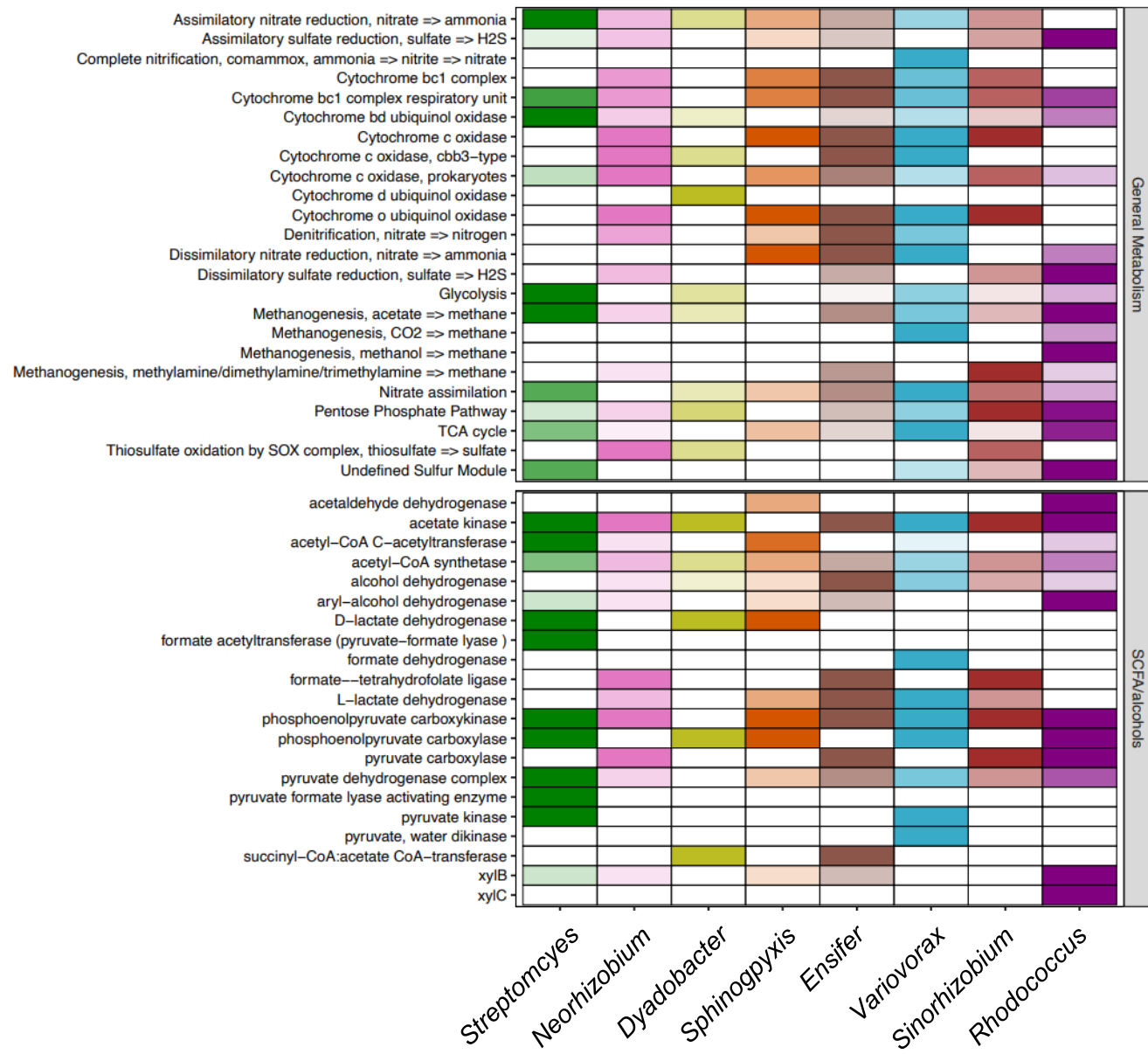

Supplement: FIG S1 [file msystems.00372-22-s0001.pdf]

**A**

### DNA Concentration

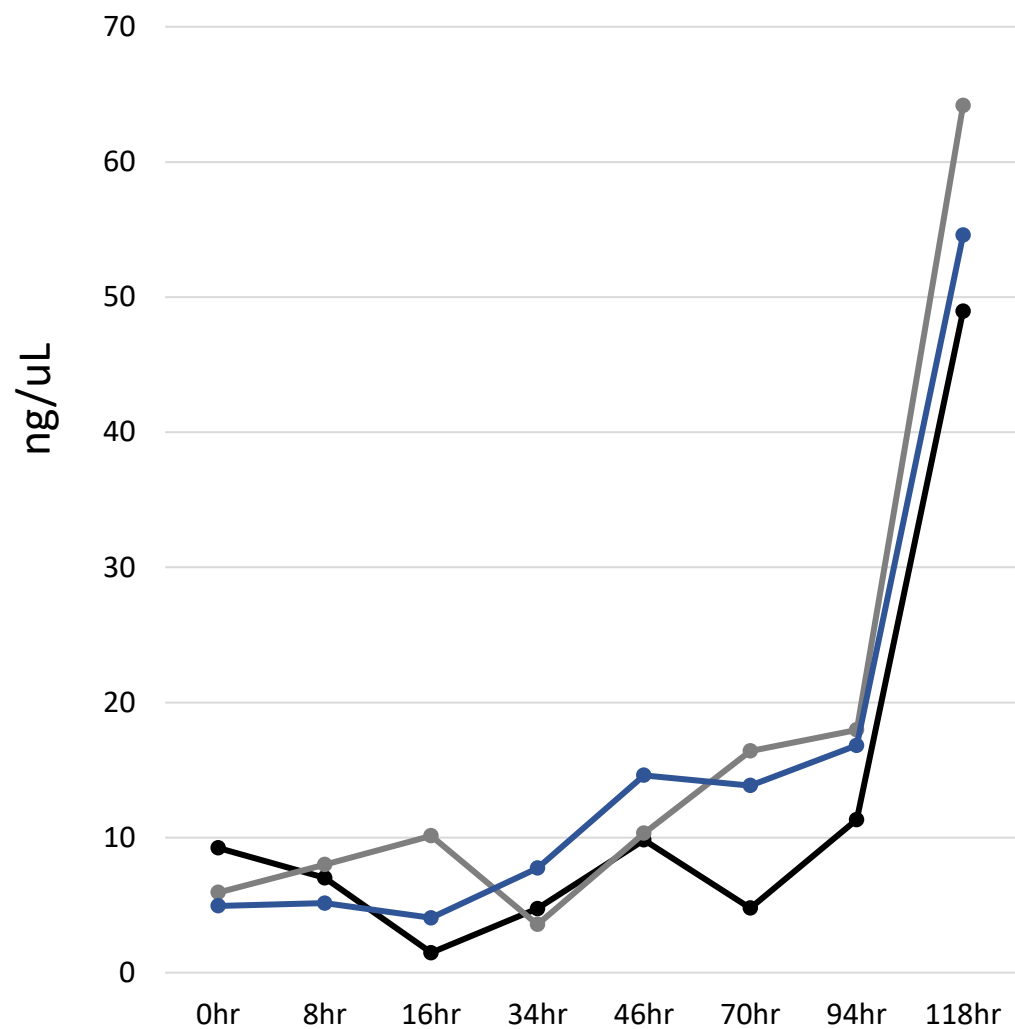**B**

### RNA Concentration

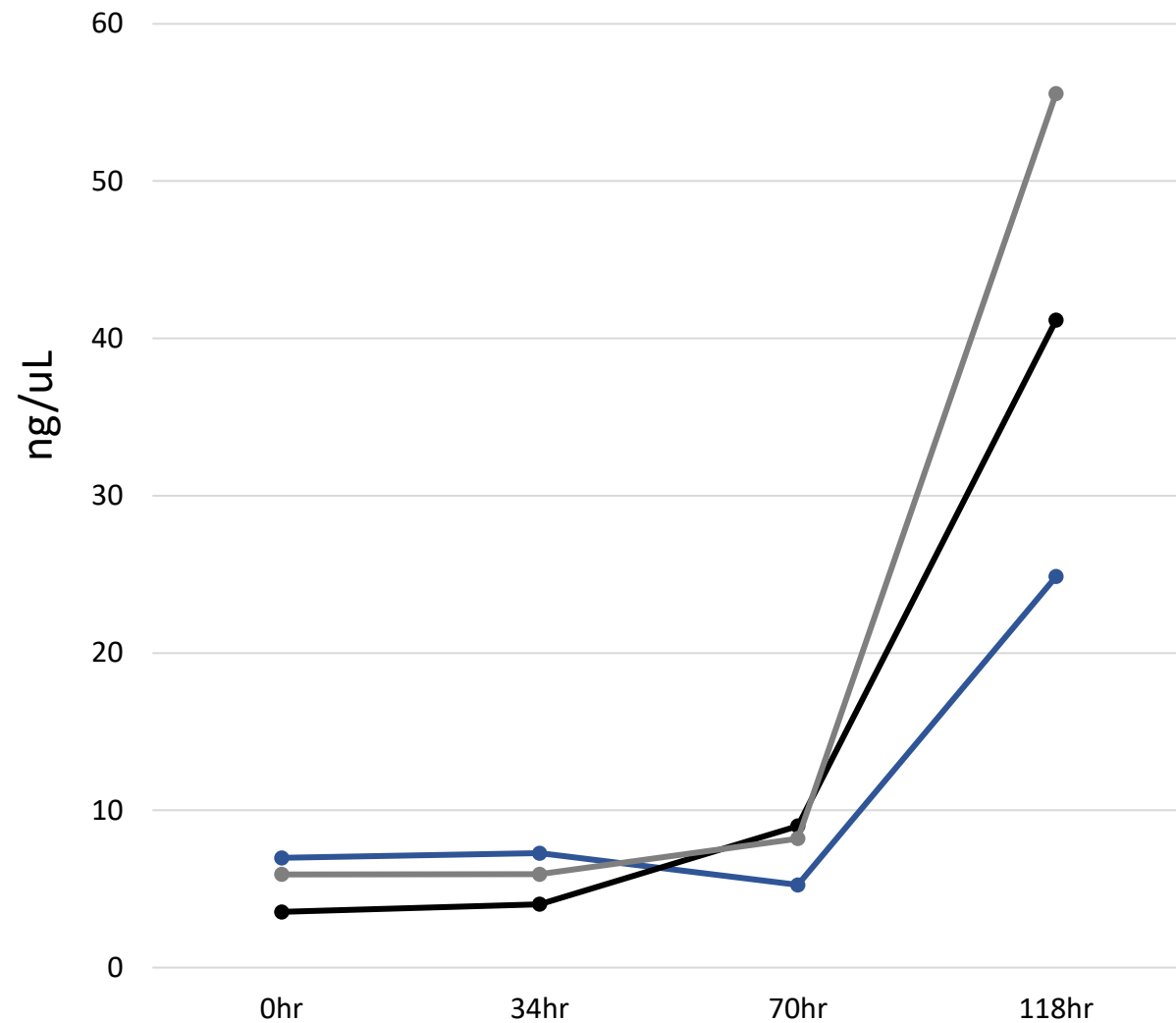

Supplement: FIG S2 [file msystems.00372-22-s0002.pdf]

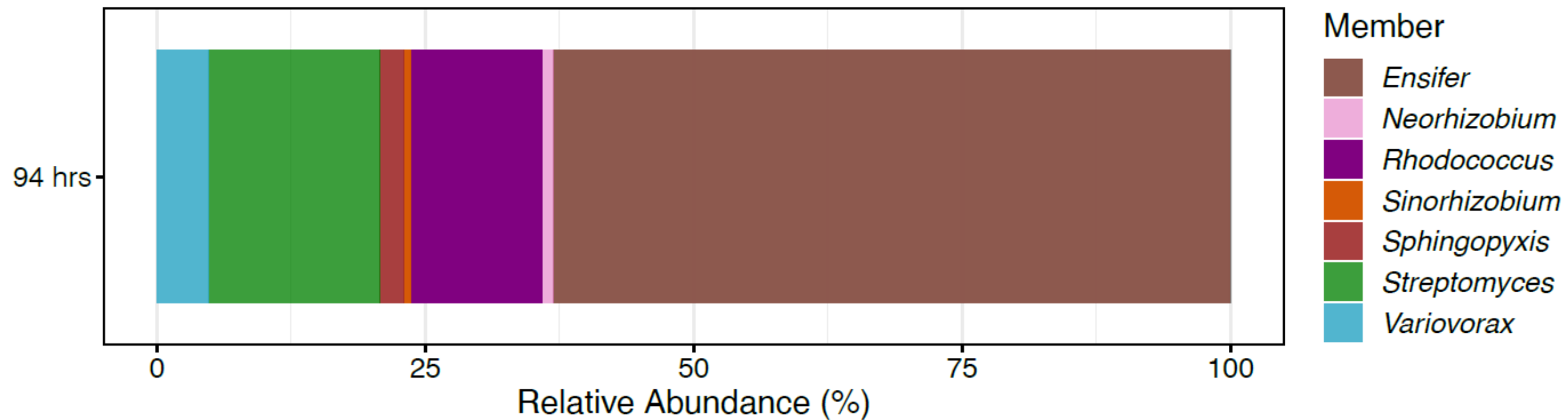

Supplement: FIG S3 [file msystems.00372-22-s0003.pdf]

*Streptomyces*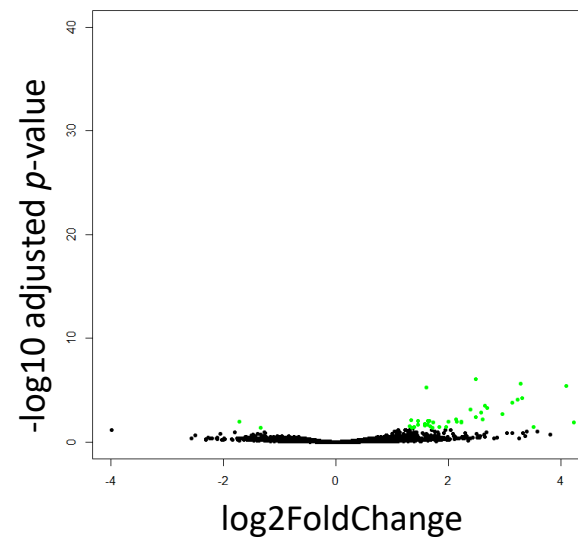*Neorhizobium*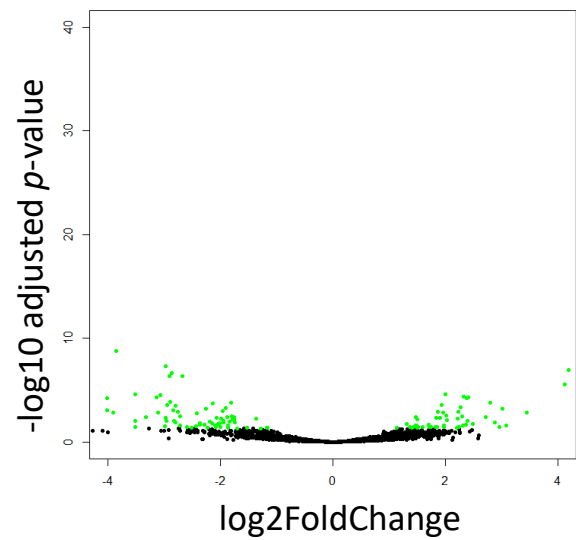*Sphingopyxix*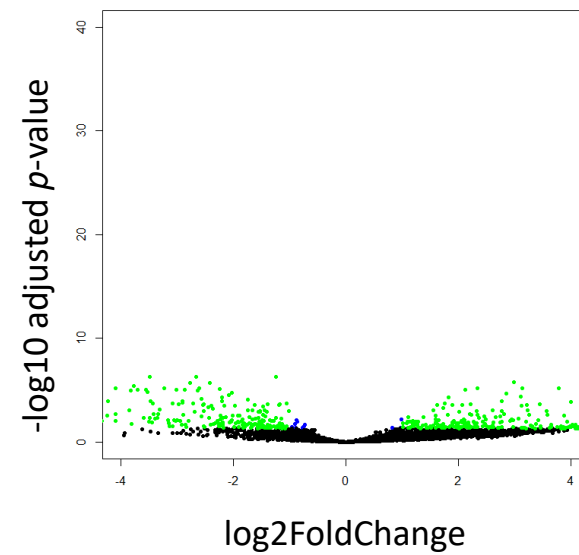*Ensifer*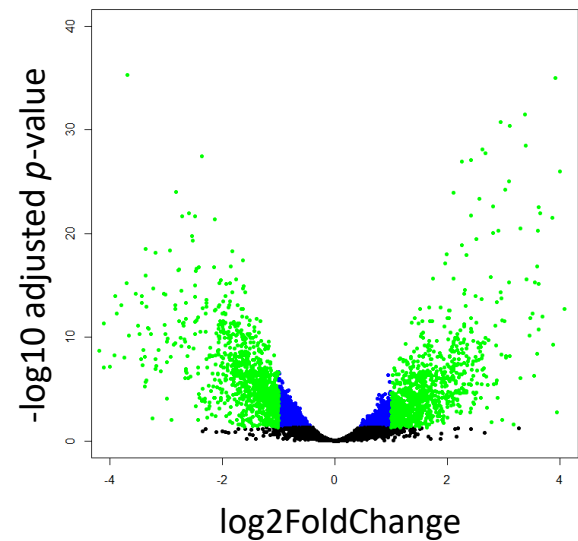*Variovorax*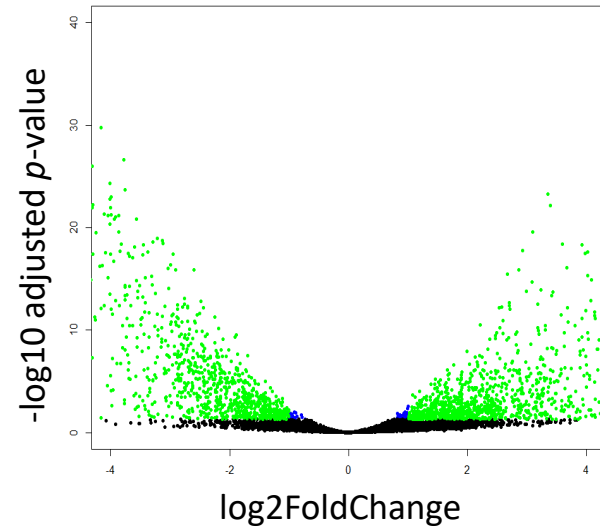*Sinorhizobium*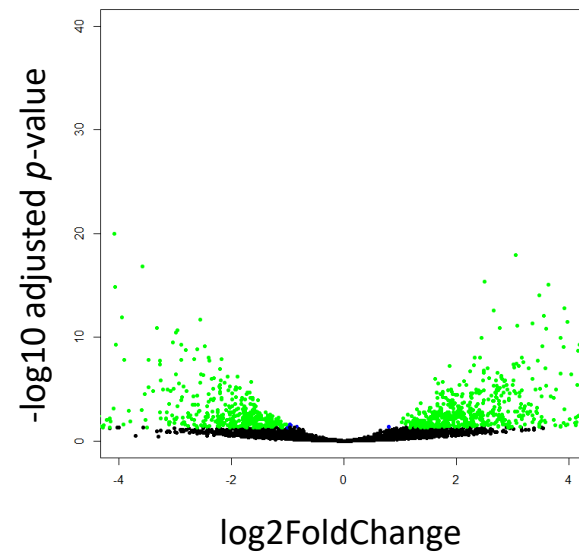*Rhodococcus*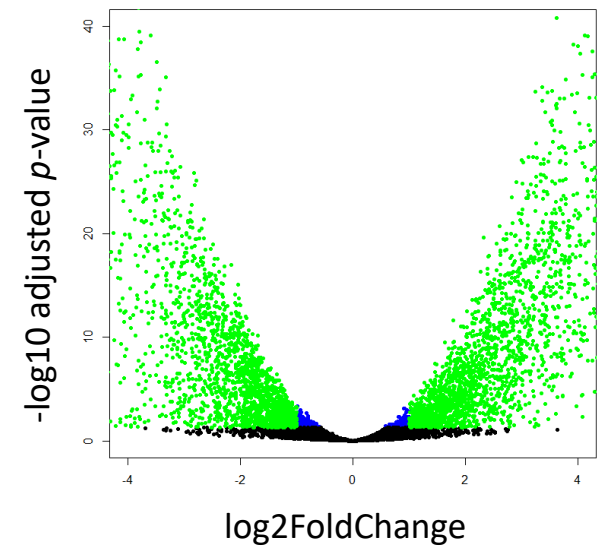

Supplement: FIG S4 [file msystems.00372-22-s0004.pdf]

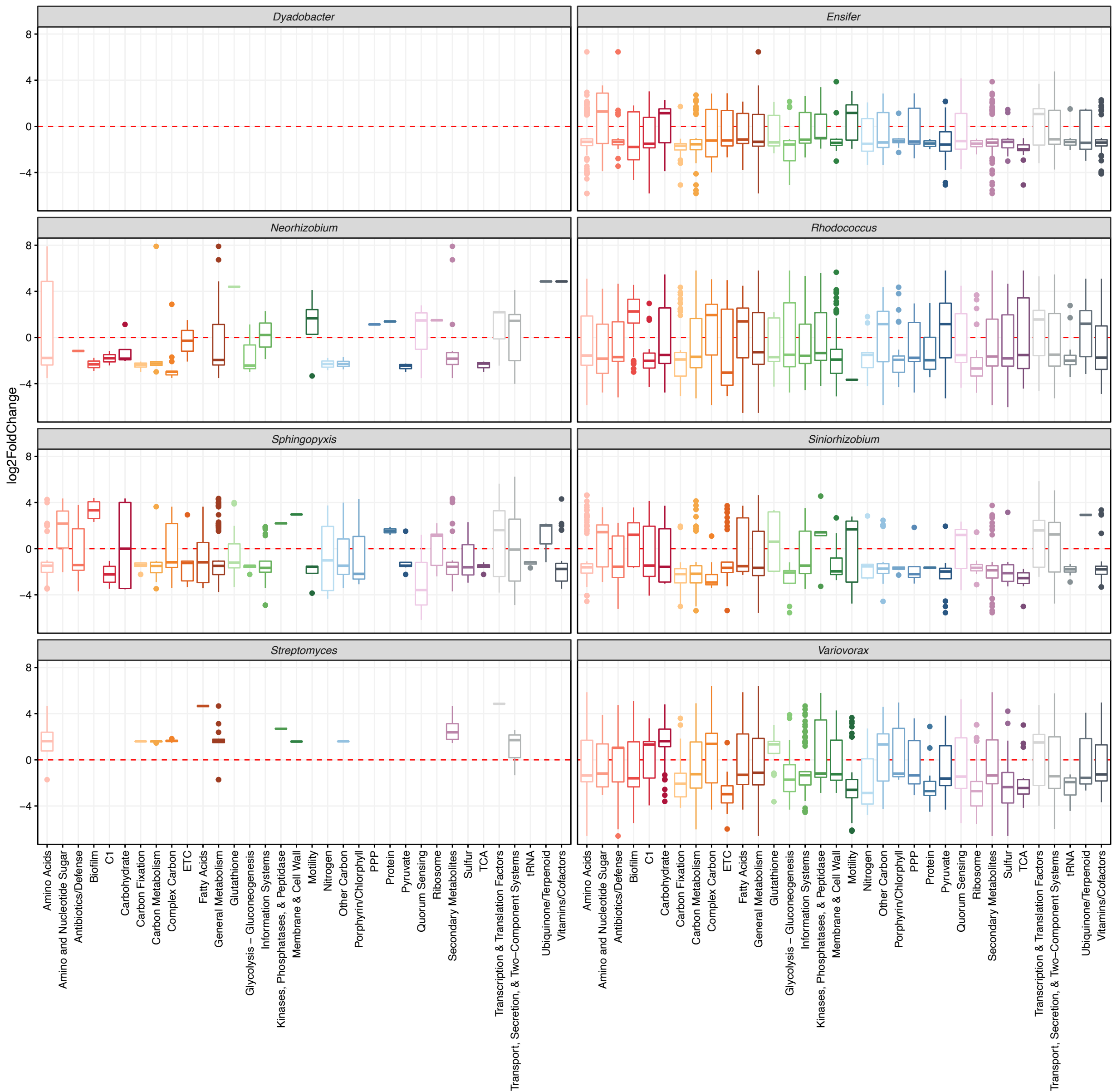

Supplement: FIG S5 [file msystems.00372-22-s0005.pdf]

Scores Plot

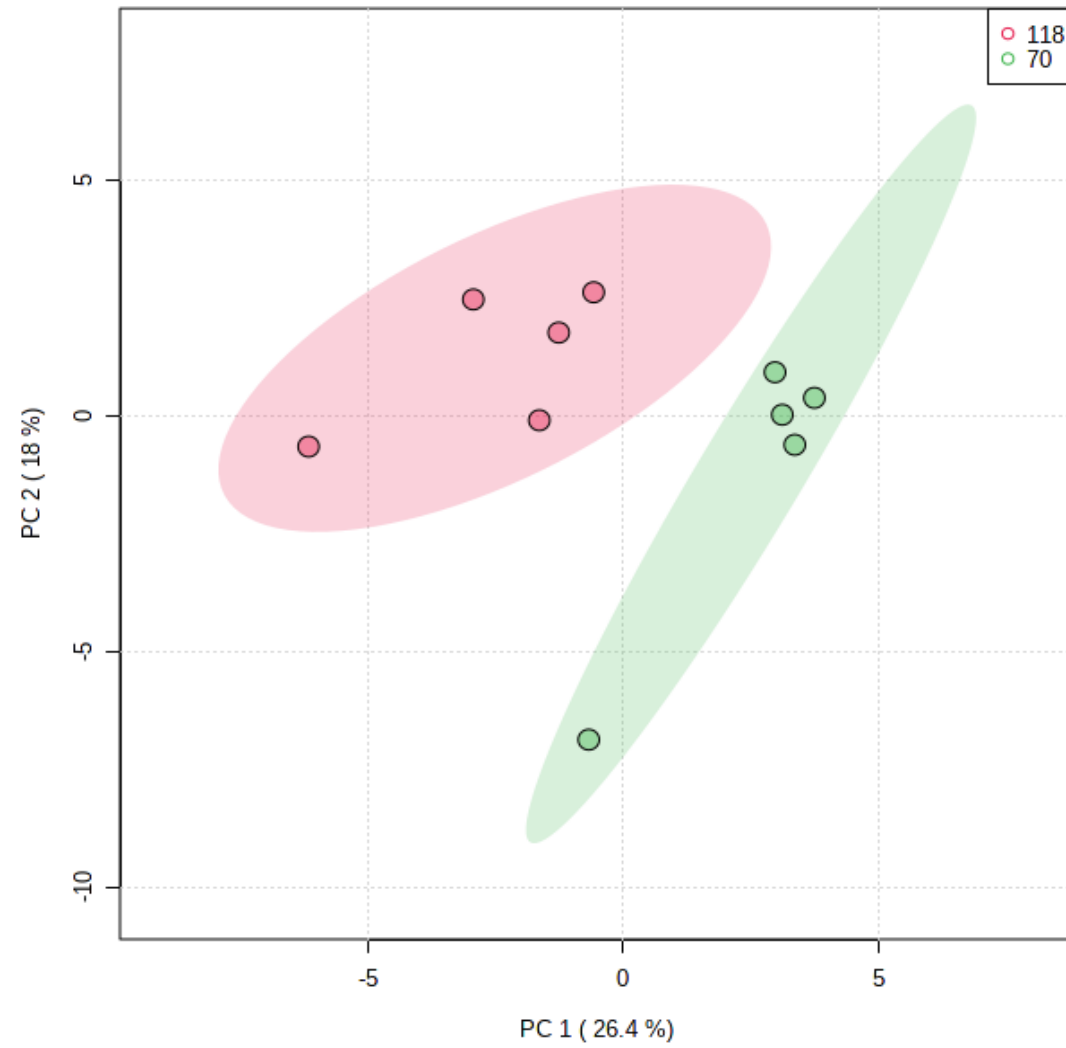

Supplement: FIG S6 [file msystems.00372-22-s0006.pdf]
