# Supplementary material for: Interaction Networks Are Driven by Community-Responsive Phenotypes in a Chitin-Degrading Consortium of Soil Microbes
Source: mSystems. 2022 Sep 26;7(5):e00372-22. doi: 10.1128/msystems.00372-22 (PMC9599572; doi:10.1128/msystems.00372-22)
Supplement: TABLE S3 [file msystems.00372-22-s0009.pdf]

Supplementary Table 3. List of Metabolites showing statistically significant fold change 70hr vs. 118 hr		
**Metabolite**	**Abundance Fold Change**	***p*-value**
Unknown 105	6.64	0.0009
N-acetyl-D-glucosamine	6.55	0.0003
Unknown 151	6.36	0.0029
Unknown 152	3.79	0.0053
Unknown 150	3.6	0.022
trehalose	3.48	0.0113
Unknown 122	3.11	0.0154
Unknown 142	3.1	0.0215
L-pyroglutamic acid	2.92	0.0201
Unknown 093	2.64	0.03
Unknown 034	2.48	0.0494
D-ribose	2.39	0.0483
putrescine	-2.67	0.0291
Unknown 020	-5.17	0.0044
