# Supplementary material for: Interaction Networks Are Driven by Community-Responsive Phenotypes in a Chitin-Degrading Consortium of Soil Microbes
Source: mSystems. 2022 Sep 26;7(5):e00372-22. doi: 10.1128/msystems.00372-22 (PMC9599572; doi:10.1128/msystems.00372-22)
Supplement: TABLE S4 [file msystems.00372-22-s0010.pdf]

Supplementary Table 4. List of all detected metabolites and their abundance in all samples

Metabolite Name	Score	Quantification Ions	Avg. RI	Avg. RT (Min)
phosphoric acid	0.82	302 315 316	960.731404	10.08286984
putrescine	0.89	174 175 214	1428.658319	15.89519328
heptadecanoic acid	0.96	73 75 117	1817.772196	19.8249615
arachidic acid	0.96	69 73 117	2113.837344	22.37643941
myristic acid	1	73 81 285	1525.441478	16.9304346
succinic acid	0.92	73 147 148	994.4037277	10.55046895
capric acid	0.77	83 117 229	1135.716282	12.39399879
caprylic acid	0.85	117 179 201	941.4028559	9.814459136
lauric acid	0.94	73 75 117	1331.415065	14.77994741
oleic acid	0.94	73 75 98	1899.160178	20.54789001
stearic acid	1	75 117 341	1916.804506	20.70461571
porphine	0.81	134 184 285	1021.465421	10.90747136
glycerol 3-phosphate	0.79	73 211 256	1459.70507	16.22728493
carbonate ion	0.99	59 72 133	822.1382851	8.158261944
palmitic acid	1	75 117 313	1720.648204	18.89772484
D-ribose	0.83	73 75 169	1378.743559	15.3380095
D-fructose-6-phosphate	0.75	57 71 73	2029.181812	21.68272976
glycolic acid	0.94	74 133 148	755.7613751	7.236502438
L-glutamic acid	0.96	56 174 248	1213.637134	13.39119843
L-pyroglutamic acid	0.93	156 157 258	1209.233414	13.33927307
lactic acid	0.93	75 117 191	742.215398	7.04839285
N-acetyl-D-mannosamine	0.94	117 205 319	1789.681586	19.56705526
trehalose	0.98	191 361 362	2480.801483	25.16742839
NIST Pentadecanoic acid	0.83	73 75 117	1622.058267	17.94182147
Unknown carbohydrate 001	0.95	57 71 85	2042.219646	21.78956828
Unknown 093	0.62	73 74 221	1562.534374	17.32719886
Unknown 073	0.88	239 342 343	1292.77453	14.32432721
Unknown 049	0.74	87 128 228	1080.122316	11.67066108
Unknown 023	0.72	84 141 143	816.2558215	8.076573647
Unknown 041	0.68	103 170 239	979.8017355	10.34769475
Unknown 050	0.6	74 128 174	1083.602404	11.7159408
Unknown 030	0.79	77 105 179	929.394952	9.647708384
Unknown 111	0.63	57 71 73	1866.552706	20.25825424
Unknown 044	0.63	55 83 147	1029.009269	11.00562498
Unknown 081	0.67	143 204 217	1400.006087	15.58871338
Unknown 135	0.77	57 149 167	2228.435452	23.29772481
Unknown 028	0.69	148 155 245	867.255251	8.784789937
Unknown 052	0.65	103 143 170	1086.545511	11.75423381
Unknown 107	0.91	73 319 367	1799.697389	19.66401698
Unknown 062	0.68	103 116 117	1185.95651	13.04767856
Unknown 051	0.61	131 191 221	1086.318255	11.75127697
Unknown 091	0.96	273 363 375	1516.787455	16.83786682
Unknown 096	0.72	174 176 319	1606.843718	17.79430501
Unknown 092	0.62	81 171 274	1540.614552	17.09273343
Unknown 042	0.91	73 136 180	982.8781729	10.39041646

Unknown 105		103 132 217	1783.939647	19.51138285
Unknown 109		57 75 327	1828.776065	19.92270333
Unknown 115		55 73 75	1892.672504	20.49026326
Unknown 002		50 73 117	683.111009	6.22762502
Unknown 010		115 130 148	734.9761366	6.947863043
Unknown 014		143 144 158	762.7752565	7.33390245
Unknown 019		143 144 145	793.3181845	7.758044434
Unknown 032		207 314 406	943.4707139	9.843174962
Unknown 035		73 211 299	963.1067743	10.11585601
Unknown 040		73 299 314	973.2028909	10.25605825
Unknown 043		57 228 301	1003.608221	10.67512985
Unknown 045		73 103 147	1030.773135	11.0285748
Unknown 046		191 207 358	1043.705071	11.19683329
Unknown 047		57 145 201	1069.713755	11.53523443
Unknown 048		55 83 89	1077.96817	11.64263331
Unknown 053		57 114 145	1089.054974	11.78688464
Unknown 054		57 129 143	1093.359015	11.84288487
Unknown 055		59 97 114	1102.073563	11.95627058
Unknown 056		73 208 373	1105.596837	12.00211219
Unknown 057		73 101 200	1119.947124	12.18882497
Unknown 058		110 180 254	1142.137921	12.47755127
Unknown 059		174 350 351	1147.436446	12.54649082
Unknown 060		177 205 255	1156.012655	12.65807658
Unknown 061		245 258 276	1180.43542	12.9758432
Unknown 063		57 83 159	1193.442618	13.14508093
Unknown 064		57 117 173	1222.586284	13.49672009
Unknown 065		263 264 279	1230.049268	13.584718
Unknown 066		448 449 450	1237.71623	13.67512105
Unknown 067		69 73 83	1241.377606	13.71829325
Unknown 068		207 221 222	1249.036794	13.80860465
Unknown 069		57 129 143	1251.763895	13.84076059
Unknown 070		117 201 313	1260.06253	13.93861186
Unknown 071		153 245 371	1268.967144	14.04360838
Unknown 072		55 81 110	1283.296308	14.21256714
Unknown 074		84 186 229	1320.431152	14.65043335
Unknown 075		221 327 591	1339.348502	14.87349254
Unknown 076		73 282 402	1342.85901	14.91488582
Unknown 077		521 522 523	1356.734594	15.0784963
Unknown 078		57 279 294	1371.120986	15.24812984
Unknown 079		74 277 292	1386.283248	15.42691185
Unknown 080		57 73 277	1386.286083	15.42694528
Unknown 082		221 281 295	1406.434834	15.65747971
Unknown 083		57 69 131	1417.481015	15.77563522
Unknown 084		205 218 576	1439.609154	16.01232892
Unknown 085		75 103 117	1447.788478	16.09981907
Unknown 086		69 84 85	1455.309859	16.18027155
Unknown 087		73 217 218	1464.469729	16.27825012

Unknown 088		73 103 333	1471.114883	16.34933004
Unknown 090		57 69 71	1472.12384	16.36012234
Unknown 089		57 69 71	1471.200508	16.35024592
Unknown 094		73 117 299	1585.84986	17.57659302
Unknown 095		57 73 117	1593.571543	17.65918803
Unknown 097		57 69 71	1631.296957	18.0313975
Unknown 098		57 71 85	1643.811124	18.15273173
Unknown 099		69 83 153	1659.280492	18.30271886
Unknown 100		55 57 69	1666.004484	18.367913
Unknown 101		57 71 99	1682.082363	18.52380009
Unknown 102		57 69 85	1690.971126	18.60998332
Unknown 103		73 293 294	1694.616445	18.64532742
Unknown 104		59 89 117	1707.284743	18.76815607
Unknown 106		75 131 173	1797.744696	19.64523315
Unknown 108		117 174 355	1805.10413	19.71243744
Unknown 110		57 71 85	1856.483746	20.16881673
Unknown 112		57 85 113	1872.191374	20.30833969
Unknown 113		57 71 113	1878.982215	20.36865932
Unknown 114		55 69 83	1887.504681	20.4443601
Unknown 116		57 70 112	1903.531325	20.58671672
Unknown 117		57 85 111	1927.471212	20.79936269
Unknown 118		57 104 127	1933.172604	20.85000529
Unknown 119		57 69 71	1941.866996	20.92723321
Unknown 120		57 69 153	1954.793887	21.04205627
Unknown 121		73 85 117	1966.695214	21.14776978
Unknown 122		73 147 204	2018.320646	21.59372796
Unknown 123		57 71 85	2062.941795	21.9593759
Unknown 124		57 71 85	2075.272285	22.06041809
Unknown 125		57 71 239	2084.933623	22.13958791
Unknown 126		221 429 503	2092.970233	22.2054439
Unknown 127		69 71 83	2118.070135	22.41112501
Unknown 128		55 69 111	2128.249325	22.49453837
Unknown 129		57 69 71	2142.320297	22.60984294
Unknown 130		57 69 71	2155.18491	22.71526199
Unknown 131		57 69 71	2163.690027	22.78495716
Unknown 132		55 69 83	2173.290315	22.86362671
Unknown 133		57 69 71	2191.894813	23.01608124
Unknown 134		57 71 85	2203.90048	23.1120216
Unknown 136		57 371 372	2268.186814	23.59859965
Unknown 138		73 147 221	2299.673976	23.83692342
Unknown 139		57 69 85	2327.73769	24.04933539
Unknown 140		69 73 217	2335.815975	24.11047928
Unknown 141		83 97 125	2336.64187	24.11673041
Unknown 142		73 257 258	2352.754209	24.2386834
Unknown 143		55 69 71	2363.650483	24.32115641
Unknown 145		57 399 400	2460.182511	25.02168315
Unknown 146		73 147 221	2510.623836	25.37822774

Unknown 147		73 112 201	2578.016576	25.85459344
Unknown 148		57 558 559	2592.040463	25.95372131
Unknown 149		73 217 259	2693.35405	26.62605794
Unknown 150		73 173 217	2754.501719	27.02959188
Unknown 151		117 131 449	2777.438084	27.18095662
Unknown 152		143 159 330	2862.133383	27.73988925
Unknown 003		73 74 295	697.6598885	6.429661662
Unknown 004		208 211 299	699.6411569	6.457175039
Unknown 005		57 163 207	701.4441922	6.482213338
Unknown 006		207 208 295	719.0494321	6.72669289
Unknown 007		93 123 125	721.8164538	6.765117827
Unknown 008		77 180 272	727.5115031	6.844203549
Unknown 009		93 95 228	731.0828271	6.893797629
Unknown 011		77 134 199	738.9337523	7.002821459
Unknown 012		56 110 131	744.8586196	7.085098606
Unknown 013		75 119 175	748.9515536	7.141936154
Unknown 015		207 223 311	772.7173276	7.471965499
Unknown 016		75 83 201	777.1255149	7.533180891
Unknown 017		69 84 85	781.3081678	7.591264343
Unknown 020		72 146 249	796.4768681	7.801908281
Unknown 021		155 225 240	798.5862865	7.831201245
Unknown 022		70 71 258	811.5849713	8.011710721
Unknown 024		267 355 356	824.0937264	8.185416667
Unknown 025		131 155 207	825.4541257	8.204308192
Unknown 026		75 129 187	845.2712283	8.479503326
Unknown 027		281 282 369	856.5657506	8.636347525
Unknown 029		50 152 226	877.6315883	8.928883531
Unknown 031		73 75 117	933.2538134	9.701295425
Unknown 033		50 85 130	956.3097245	10.02146708
Unknown 034		59 75 201	959.0131195	10.05900845
Unknown 037		73 133 211	969.3781195	10.20294461
Unknown 036		73 74 75	966.6613192	10.16521708
Unknown 039		73 74 75	972.9778562	10.25293325
Unknown 038		118 219 295	971.282313	10.22938766
Unknown 018	0.74	71 148 204	784.3647762	7.633710698
Unknown 137	0.86	57 71 85	2286.500819	23.73721686
Unknown 144	0.77	73 147 221	2372.907898	24.39122503

Avg.S/N	Hits	MetSuprnt_RepA_70_hr_SFA_2.cmp	MetSuprnt_RepA_118_hr_SFA_2.cmp
219.0048885	11	58081229.46	56722361.56
81.88497376	10	1864131.805	733071.1678
48.5394481	14	1776598.976	1752473.649
13.20142779	9	1029837.207	949401.9841
307.3835233	14	38648888.41	37806536.62
47.22810043	14	1470847.538	1239739.829
33.94582387	14	425943.5815	385864.729
44.37174172	14	1619002.059	1475389.362
54.01183803	14	2028732.788	2061291.166
34.09632665	14	2664619.513	2195860.76
430.6445852	14	38066121.36	36648824.52
99.41558306	14	2080700.277	2060662.553
22.65782715	11	927257.988	1040730.015
236.4942597	14	9362367.894	9590866.865
362.9707404	14	27208900.51	27175756
38.49046589	14	1180037.158	1350346.189
18.87899591	10	977733.3543	1025097.991
26.07757148	14	304373.7501	269947.5947
97.52385729	14	1237246.2	4059617.169
110.9016092	10	2080686.564	3043484.442
71.67375817	14	1930469.403	1631142.188
62.3245663	10	435579.7627	6533702.835
314.2815561	10	6255579.847	28793806.93
29.73465791	11	1434339.384	1140252.264
16.96410088	10	849393.3414	793943.3103
39.88552089	14	787315.8147	1021346.252
15.8969029	8	0	315812.2991
6.421333014	3	0	0
20.08629649	14	180214.0123	242559.9416
30.36223765	14	486900.6476	640512.2857
12.9548591	14	109515.2337	94053.14827
36.24519345	14	640912.3525	426529.2212
14.60560674	14	821244.4251	1458759.519
28.11510505	14	450073.1997	595948.8046
7.959859795	7	139850.189	135559.9279
27.9312601	12	468938.8667	584680.5999
30.47537877	14	389649.0679	292781.0057
11.53950388	1	0	0
12.02381939	14	655702.2145	4375531.601
122.5165287	14	3411786.19	3610750.141
21.04285532	14	174369.3217	195740.3193
19.32425388	10	116506.0735	70098.08453
32.48805213	10	199644.4011	156527.7196
22.15327001	9	483785.1415	181196.5816
6.368751464	2	0	0

91.16693455	10	1239380.169	7386720.482
28.78325645	14	435001.7619	330975.2926
6.740046821	14	3358060.242	2501457.592
7.603979755	2	0	4215571.832
434.0675415	14	45160865.47	45290138.93
74.50788074	14	1857054.598	1732490.822
25.42771781	14	60900.92584	628704.3645
95.96991281	14	2776796.449	2682616.332
271.3517789	14	186127542	444525918.3
76.51278074	2	236397394.1	0
17.70015298	14	99891.8271	125145.9198
9.357534536	14	487864.3048	559069.8455
19.42992754	13	275435.5813	183129.5784
60.30528833	14	824752.3303	772414.0968
23.22367408	14	255254.9439	248583.4739
83.57173215	14	1813918.626	1877684.551
39.5067598	14	996874.5825	1067065.25
35.35769667	14	585798.603	613722.8354
85.71028517	14	3806621.222	2015646.513
15.28211781	14	834954.6826	950221.2668
189.0451688	14	6072139.27	5872307.454
16.14789831	14	22674.3881	131050.9688
17.61354353	12	195547.3213	92653.45619
24.65200872	11	297331.7241	129625.4546
175.8775871	14	5185041.038	5505717.235
89.19717368	14	2072573.918	2158275.462
127.0732389	11	8903498.819	4593386.326
94.62599455	12	1825381.653	2784770.45
6.629388014	14	2296578	3545024.973
12.7809469	11	123947.872	15272.54193
37.96043201	14	423921.2744	545988.6819
41.85268724	14	1236598.458	580473.4415
22.49772605	14	185220.0684	206710.9764
8.674408833	1	0	0
8.010055788	1	0	0
15.63423429	14	197539.2426	457634.6955
40.13145897	14	957920.0898	473543.4267
36.56402647	9	0	723355.1837
23.96976877	10	431959.9765	290330.3418
16.02622053	14	407626.2613	286676.3762
19.30491146	14	833592.6036	735529.3783
30.40556658	14	239295.0577	326514.9733
18.47193688	9	0	267714.2656
12.65740295	11	114919.0581	97822.18512
79.49274949	14	2520884.891	2534940.689
11.94586458	10	86788.45405	253194.5655
11.60242509	11	987703.4402	1076478.167

14.94988353	12	510494.591	648062.4759
7.591806148	3	0	0
8.50151199	14	64446.40328	226411.3998
6.600703683	10	672678.4867	343560.3709
13.82603944	10	272691.0606	449386.8622
8.989390886	10	372761.606	330319.8132
29.20632199	11	1004635.369	1222472.446
11.64964355	10	441592.3348	435966.5185
22.06684811	10	573119.5324	963151.8709
15.56112567	10	701587.8502	778189.4672
19.41429327	10	788339.7057	812525.5428
8.350416612	3	0	0
46.5634551	14	1266147.067	1353656.496
8.958235055	2	0	3121559.261
7.624633255	11	237036.6551	388317.0066
9.47572384	12	342458.4377	1220520.491
14.97547814	14	1025288.625	831554.0324
16.77098993	14	1664100.608	1551506.838
6.686505886	14	636696.761	369675.771
9.737680619	1	0	0
13.7339682	10	679656.7961	451594.8102
11.69003178	10	380968.3202	443786.3775
17.52998604	10	798590.553	829025.8657
25.98161808	10	740123.118	641417.1238
40.03108076	10	1431600.034	1438903.729
28.76534885	12	0	357774.8301
17.82605282	11	862330.51	818626.2972
15.88279566	11	1122678.034	995256.6315
6.094191464	12	564302.5976	442980.9301
33.30645255	14	340497.1251	388935.4258
7.094871807	10	1425134.201	1168752.023
23.8670767	10	1085432.729	953743.619
9.169765181	9	670128.7651	621042.9951
11.13525387	10	909297.7796	751892.0539
10.76678552	10	919713.8419	474757.5589
9.416087195	10	557088.7983	386812.316
12.17618876	8	477693.7473	0
13.59801411	9	562744.9723	636293.8085
41.61122085	10	683265.4723	740976.4367
9.415320369	6	0	0
15.60868314	10	1297671.567	1239420.595
27.86460321	10	727712.9861	1746753.743
6.398631905	10	514224.5219	644293.3109
16.74621796	6	0	502826.8278
6.88832506	10	820069.7599	719552.9752
93.69768873	10	2251277.37	2693362.515
11.65423495	6	541912.123	594313.9074

19.84865417	13	534419.8916	463121.9034
87.32475844	13	4375017.363	3658827.818
6.842914417	2	0	0
8.63396036	4	0	290830.2435
24.18178096	5	0	525948.5208
16.75803302	10	86534.31565	290407.1108
18.68545815	14	3041605.088	3171901.243
17.69075263	14	234332.1032	151239.9199
9.323013431	14	591756.7371	365661.2441
385.3196854	14	39927370.08	37322845.55
490.1184739	14	56242822.2	56434053.44
46.74362701	14	620797.1407	577825.6585
54.91892785	14	1011153.819	936602.4794
90.88360038	14	2164310.106	2047181.245
47.58915116	14	1184079.731	1410463.219
31.83952856	14	1039792.53	994661.0101
138.7883528	14	3795110.971	3373125.906
70.94860539	14	1730039.45	1870758.371
113.0325325	14	4355492.099	4223228.072
30.8478166	14	3431621.397	58975.22729
297.1443207	14	14556384.08	13140532.29
89.3562423	14	2245599.555	2172736.154
21.53968003	2	0	0
13.08642269	14	241236.4206	87058.74562
18.80878655	14	386016.845	2829661.591
234.084694	14	8722659.754	10383958.33
6.41998621	3	0	260788.543
52.96846873	14	2688422.49	2138868.008
7.888714345	2	0	1623380.594
21.90387782	14	15842540.52	22980271.31
64.32177395	3	48371264.73	0
22.26250539	1	0	0
42.55953786	2	0	0
8.365165369	14	16207.81106	135061.2269
157.2334042	14	3935074.883	3995438.487
7.012493374	8	892432.8443	601225.5614
47.33179496	14	788380.2528	877209.8347

MetSuprnt_RepB_70_hr_SFA_2.cmp	MetSuprnt_RepB_118_hr_SFA_2.cmp
67766127.38	58338081.14
6564944.793	1589521.041
1428085.192	2032702.698
0	342677.0434
28875660.2	41588983.33
1123319.27	1219713.739
460022.4378	427085.1183
1312034.828	1463672.373
1986920.819	1585614.381
1476501.205	2601557.598
35077429.03	37457183.75
2146918.229	2217513.054
1026797.417	1065208.91
10484918.52	9695865.013
24429892.16	27208728.76
1282731.762	1639550.892
638845.6419	1102695.699
314710.1861	291758.8367
2722673.643	7907609.845
3466827.313	9642243.769
1569877.77	2139392.596
878568.1497	2241407.764
26504088.43	50504048.76
843434.0803	1995169.279
207684.8892	427405.2577
814434.982	892454.0466
319443.3297	242249.02
0	437556.0937
256636.9038	210378.8415
421753.5241	603534.6266
289126	265596.8584
1100086.34	670064.1113
903822.2792	1130253.363
883709.8051	614489.7466
0	13471.30357
626411.8804	398764.1935
599851.6691	311030.2726
0	0
1188933.118	1908244.835
3714968.016	3587764.071
222529.6744	147080.8191
270865.2389	182699.0389
1903109.437	461418.1958
0	468906.5815
0	0

411663.6568	5560088.111
317340.8999	447304.167
1875094.168	471919.57
0	0
45057780.26	44596318.52
1788936.41	1633520.762
43980.86185	784249.1412
1553194.217	2657329.56
364764412.9	190112511.4
0	0
125989.375	126729.6937
1466243.789	787764.1613
179353.583	318327.8374
602675.4274	871338.4769
255227.9408	308078.0255
1741737.102	1863966.28
728585.2455	986398.2649
613824.8497	601404.6634
2835500.287	4169301.17
942349.3188	1449529.957
6024430.556	5722581.449
24668.05758	244829.8458
119778.6993	138895.5791
278486.8308	221992.528
5371981.503	5423594.296
2284664.553	2129089.887
5335667.738	6172701.494
4448696.707	1931210.596
4309751.39	2560196.454
506099.0859	0
513956.9078	583499.8683
809409.5972	1350496.127
226007.9802	209109.5298
0	0
0	0
293881.965	235982.1249
1037853.344	410507.528
587931.8761	525380.7696
367250.3339	0
327582.0058	281205.8811
827755.5266	734802.2328
293726.3134	237987.8241
598085.8843	205203.284
81297.14891	104801.9664
2394808.988	2611516.452
737728.5021	107394.4886
1206834.324	607693.5882

1031519.417	614362.9627
813408.4625	0
813408.4625	81724.50694
262980.9455	277146.2428
455044.8777	392707.8726
501920.6261	149153.3055
1622763.008	803187.8833
325573.8946	381996.6923
854060.1117	840217.0084
678685.034	596723.1563
692079.8346	845355.9109
0	377466.9181
1575918.968	1577709.143
0	1169557.572
396986.2062	232085.4991
0	920358.6243
351836.4683	1253968.424
463728.3165	1487546.424
394843.3523	770601.9969
0	0
0	462442.5334
0	403251.832
0	775920.5773
479273.8293	756455.6052
1553551.744	2202493.305
495480.0084	1346327.946
682975.8445	728972.8129
786140.2789	1001080.004
602709.6249	501257.0053
463897.5479	297596.3065
746621.8405	938466.8255
596033.1176	929826.706
0	814982.8104
173218.1633	535168.0189
173218.1633	531056.2596
412417.7161	325249.5151
343918.4091	468188.587
463215.2303	602595.7442
1077471.543	1524808.681
318950.3399	0
850932.9268	844443.2182
603331.2564	801606.7248
417289.142	476409.2395
544078.5531	161572.8076
490235.5302	734944.3899
3809042.902	5028432.388
996259.9782	0

466617.9544	483760.6717
3040019.459	2797911.543
0	0
0	0
0	185639.0478
79866.16387	355938.6679
2950660.212	3739702.205
416812.9527	389419.1445
730308.5587	1941092.506
39229354.12	37963611.57
56045398.31	58497796.71
536461.8332	601603.0973
990952.2753	978807.4713
1883814.345	1942684.961
1186490.041	1286096.384
1852268.777	1966886.275
3596639.381	3597571.393
1763489.401	1735866.103
4895765.638	4334757.168
2862697.097	78485.45328
13495789.15	14153200.56
2060219.702	2399765.306
0	0
175068.9044	362142.3698
2576351.161	285543.8113
11401635.64	9090901.902
0	0
1581910.578	2946909.136
0	0
272650.1877	17504934.96
0	0
0	189324925.5
248259505.9	0
1085387.9	135139.7931
4079010.368	4152337.326
562561.885	573774.6465
1846445.273	740697.8712

MetSuprnt_RepC_70_hr_SFA_2.cmp	MetSuprnt_RepC_118_hr_SFA_2.cmp
54963463.61	62658551.28
4607031.125	3118591.143
2396753.77	2151706.469
701183.8747	846112.8159
42189054.59	31688125.12
1936067.877	1577130.575
417802.3689	385513.843
1892062.502	1556048.705
2339507.372	1323825.236
2025689.048	2151458.075
37231112.64	33052486.65
1729803.949	2666730.279
1279323.402	2125457.705
8121026.267	11624430.7
28164795.5	25193177.53
1296233.675	2584932.721
1056225.024	1411185.221
322111.0011	302708.8976
5819149.123	18637849.03
2570105.146	12369466.61
2182412.343	1579721.694
709389.402	7651891.636
20004249.46	83001721.32
869888.2266	1089608.457
992999.2046	157512.1542
986663.0828	1203932.527
119472.2195	270079.3773
0	40579.90814
419102.2062	242052.225
678704.5592	462622.9343
818421.5209	247699.1238
833808.746	552802.1868
1259854.036	1771522.092
964275.3742	814537.4053
0	39417.2371
452196.4343	0
747227.9563	529323.0794
5259973.456	0
1070220.57	956940.1225
2961741.717	3997118.864
545322.6551	319211.5824
388969.6863	346520.3459
982386.2716	909217.2686
500309.5085	473049.6188
0	839106.3988

1868093.08	13656278.65
1116546.549	409407.8505
2118632.252	2642792.834
0	0
41905121.2	43852080.6
1726900.925	1635843.072
1860176.029	728588.7415
2314068.411	2479769.695
291084027.1	533255947.5
0	0
97486.84752	163606.3545
1020049.575	1425232.931
263801.6555	228503.6985
787470.1863	803257.9708
267061.2381	307614.9782
515170.397	1758775.766
779523.3207	999257.0715
558586.0617	613562.8202
3060175.307	4890437.397
322081.4585	4608160.588
4977790.901	5484396.855
218552.7092	352648.3905
102552.5146	143233.7527
431876.8751	391878.5734
5166871.91	5471752.172
2272795.337	1968860.794
5889405.148	4810574.678
2641079.769	2779429.81
3304118.174	3445363.601
30126.74455	132904.1943
667766.6991	488075.036
841122.3164	629340.8277
137589.43	205569.8388
4124206.551	0
3756342.895	0
359278.3259	534364.1198
469613.4248	838291.2621
468787.7007	1173280.319
276756.0916	221602.9333
248215.0612	256593.508
738041.1547	918418.2103
209101.6341	368790.1196
238733.5364	452202.6951
77447.51871	98209.57427
1947394.078	2667829.508
155667.9426	652835.6852
1313713.972	1110016.921

860112.874	1030367.577
0	753209.311
38721.93237	951843.381
684828.7347	950977.0803
754834.8759	521350.6204
337989.2774	429078.5098
1363287.571	942761.8215
548328.3698	466352.7393
1193900.801	954376.3631
926889.1128	451381.2194
1032751.962	706543.9533
0	289482.8077
1364676.776	1782113.868
0	0
0	402710.8701
0	183039.5633
1415434.146	518409.5435
2029390.047	1260261.855
993484.7062	351543.689
6134473.21	0
569635.8992	237227.8204
456131.6088	247694.8554
928008.8923	651179.7738
872858.4132	508281.8513
1535352.012	2417055.688
0	2951736.53
927002.3431	787943.7555
856134.5208	641422.4885
876130.88	61612.08663
419133.4254	453018.7426
1443638.782	853973.4782
1166507.513	648424.2751
1116291.196	701079.3936
1063108.186	298624.7663
881953.6644	586318.5763
543635.9722	136844.1765
532843.4081	0
618920.9048	0
838085.683	857790.127
0	920855.0803
1223567.415	790072.3284
1536782.979	1775449.747
581765.5895	246864.5111
0	963250.9052
925437.9889	25745.44086
3329320.218	3414896.439
0	629177.4421

450084.9018	604448.3163
2720058.055	2634247.099
0	2243764.424
0	1572356.235
0	4297653.282
123439.2794	176231.1166
2125512	3844618.403
252013.106	261854.4888
657532.935	651127.723
34047550.71	36609605.28
55530846.73	54059387.01
538662.6142	635178.1567
490951.8784	937415.8954
1824375.328	1987930.656
4904703.645	937712.0948
963892.4663	909006.118
3103353.764	3420053.72
1534632.797	1790714.765
4199126.493	4698087.042
157405.2532	96252.3888
10898070.02	13464669.13
2116087.01	1974403.777
0	1846885.405
261498.1096	1090735.108
267216.6932	2521542.296
9350310.342	14667711.13
1374716.482	0
4654196.168	2261486.071
0	0
1211464.214	32849554.88
251786384.7	0
0	0
0	0
1191022.152	161957.2951
3687443.582	3986160.373
0	701727.738
1044047.919	1362908.892

MetSuprnt_RepD_70_hr_SFA_2.cmp	MetSuprnt_RepD_118_hr_SFA_2.cmp
57559244.64	69211371.07
2036933.012	1685022.071
1255951.147	2081627.982
430141.3974	806949.0191
32635037.67	41661587.92
1225793.623	1474570.281
410815.4837	492990.892
1697650.335	1440915.187
2266576.147	2387152.515
1955544.37	2182797.819
34362399.25	36985913.57
1801944.964	2349069.858
799855.0592	1001975.639
10623717.12	9632385.799
24509615.58	26032719.51
579383.1613	1740533.782
704814.4218	930428.3719
348166.6595	385474.6237
1304276.014	9211773.311
1530158.902	6856402.252
1170157.03	1637987.94
905035.9011	4945812.332
4753987.032	34377902.25
818203.7598	1483457.514
819079.8554	440656.9243
801466.3527	1110999.279
232669.2336	0
0	0
240023.5202	328873.1292
490447.7233	403069.6439
249175.398	198561.8058
713352.4696	1186739.206
2451976.319	1142452.347
533765.8769	565133.1904
145416.5552	90920.35515
540330.8359	674462.131
483629.1426	657935.7242
0	0
736259.6367	1092837.396
3396002.547	3750030.325
214345.4894	346352.2999
98844.09407	150167.2964
164830.9425	248844.1381
540100.3845	562998.4742
0	0

359096.9809	6963085.464
934051.1005	410786.2512
2557749.275	2748687.649
0	0
43415260.89	45293738.23
1535208.421	1809649.721
286875.7469	1365802.003
2508160.954	823604.9552
468932452	474444857.2
0	171566373.9
98304.66996	224249.398
578555.0717	1034613.262
213839.2129	290753.4082
721522.9984	811192.113
285767.9666	269023.015
1711882.507	1899589.308
983314.9182	715473.9584
546879.097	634836.6789
2924278.321	4830842.81
279908.9226	271195.0978
5424168.805	5992430.775
150656.4046	265984.846
129354.123	134177.4313
256319.0974	378239.9248
5072010.969	5501078.289
2008340.685	2161214.006
7307401.991	8223270.221
1706905.407	2823358.057
2445413	3286873.927
129231.4415	541404.0959
431343.0142	520593.17
777512.0977	533143.266
211938.8775	200694.5358
0	0
0	0
277572.7146	412966.2651
510406.2087	506933.7033
0	859058.1735
210634.0409	727595.7163
340039.7193	448197.56
714589.8363	1244673.143
347893.7147	482657.0091
270840.9298	840669.3165
86633.34871	79362.75661
1917735.95	2866358.424
161204.1612	1186896.661
880056.4762	1041984.516

639486.2235	741008.9121
0	1587698.642
8376.268774	1522037.621
432272.4863	523348.5058
360796.4525	871350.4399
201749.7062	1032510.854
921284.6064	2146915.154
392131.835	1071679
935554.789	1490454.448
801672.4377	1257643.458
724372.8721	1228958.667
0	0
1146970.118	1512259.479
0	0
210670.5961	0
1226948.968	611836.5504
1022809.863	891177.4773
1389921.404	1611718.28
722842.6206	438427.4725
0	0
544281.7801	595661.333
392461.8676	321754.2857
764898.4294	643629.2579
599147.0727	748251.9458
500698.3321	2308653.562
363018.7583	915724.8638
888288.7475	899332.2756
1068435.086	865006.9085
573912	1017344.227
395916.2299	351790.4108
1051460.721	1027498.329
789990.6931	810552.127
480199.1546	411489.6628
711145.7637	894475.3248
577725.4853	813205.3518
2205049.61	645363.4533
376092.6341	483825.6424
566947.9443	640998.0799
660912.0394	1138010.394
335156.4178	44255.76038
844124.251	1284552.855
642847.2822	758589.7845
526105.5032	442454.1377
0	350105.4659
572635.8786	750856.9631
1827796.696	3232841.952
0	298466.3088

372714.1672	672752.5078
2086852.675	3630393.848
0	0
0	712085.6671
0	1287538.374
146062.3481	406296.9616
1726376.543	4546236.851
220025.4185	171551.923
496862.6659	1754992.843
36385831.87	39854260.44
56772367	55437524.9
614781.7502	744524.1601
871093.7891	940946.4158
2167510.715	1961320.637
1181450.955	1066974.194
1959848.606	1012970.786
3540524.692	3867970.975
1617760.816	1870644.241
4241033.866	4212665.431
2440718.755	55590.9195
13684618.32	14321884.99
1981988.445	2191100.963
0	0
251587.7611	309875.0964
419090.2686	472324.9249
8142345.249	12801271.33
0	0
3051216.411	3950398.515
0	0
5362798.514	16610700.01
86374815.38	0
0	0
0	0
224224.4425	176611.5808
4033011.035	4029254.632
0	687663.3631
784687.8806	972259.9141

MetSuprnt_RepE_70_hr_SFA_2.cmp	MetSuprnt_RepE_118_hr_SFA_2.cmp
56614732.47	61276766.24
5873413.727	2053070.717
2313627.934	1978425.703
0	770718.079
44581583.25	42678573.25
1744347.539	2244588.701
448986.8118	428589.9832
1536511.233	1511021.277
2475363.657	1582265.317
358732.3323	1472987.013
40090159.53	38483064.24
1874918.183	2637087.314
893522.0648	1645990.27
10751788.67	9374652.452
28084058.7	27907916.82
1480723.914	1725053.397
1294456.689	1435090.874
331678.5821	293503.9266
10100536.87	9034783.539
6405652.644	16194643.65
2154709.83	1690888.108
570752.5497	3183972.478
20386693.85	82929858.08
964790.7662	1784419.196
937415.9948	223683.1445
817722.1835	1013703.571
238095.513	265989.1846
0	385566.689
141496.8802	327472.516
632120.2039	355555.4127
173748.7953	242333.4383
641525.7179	748555.604
1114713.133	1108879.608
674799.8932	670553.2198
0	216223.2087
668715.2846	586705.6242
418301.721	479683.7668
0	0
986257.6596	918217.9832
3471701.104	3431950.698
221498.6858	287073.3059
397626.3653	317359.4955
1414844.693	809402.4097
393741.567	274987.4869
0	1369694.775

331286.7449	7169777.173
993363.9641	516480.3898
739629.6098	397109.7538
0	3357831.767
44691882.65	45157320.18
1945782.634	1615941.916
80022.04713	1258220.943
2733702.776	2881433.124
454654754.6	456374608
0	0
126704.0487	117550.3185
993962.8077	1010699.501
423677.6556	261013.9996
950184.1236	830319.5413
313420.1312	247951.554
1867906.53	1755150.117
1087558.751	888407.6887
628957.8525	528525.3593
4056697.904	4848913.081
1069742.394	2496543.332
5856217.441	5810288.176
189304.5159	320772.3054
148421.1292	171320.821
246965.4048	470793.7352
5677276.486	5325417.774
1979279.759	1986187.832
7825034.813	6606007.264
2493714.446	2375304.394
3014287.587	2727170.491
0	165757.8699
630263.8673	607498.3819
1171768.708	972346.5544
199230.7476	166552.4843
0	0
0	0
147982.5264	237942.7497
796430.43	637893.978
531303.2602	721760.4615
311827.2944	298142.475
353688.9529	322477.2206
1143720.393	946853.6437
267647.6325	264655.4746
388777.099	180002.3124
105700.3376	130704.317
2620294.371	2308787.105
443770.907	200883.0765
553622.6543	1100771.232

462596.9943	560637.8869
0	0
613770.3371	29882.80527
410134.7772	379098.8091
399613.0335	435650.792
274239.8867	185933.0378
1566447.868	997080.3834
316734.1509	628165.4837
1299104.075	829265.4501
1042873.297	790436.8787
1081673.688	768746.5428
0	947223.7569
1272350.369	1738106.564
0	0
0	319392.7534
1501434.175	1144377.314
1416846.178	1005685.673
1981061.07	1539920.554
846541.3372	1010050.527
0	0
655387.3067	569675.7929
495117.1658	387761.4125
855371.8836	847847.488
988921.7444	716341.4875
1559614.233	2107920.76
310310.8558	3164173.678
1154336.292	1046041.478
1194138.352	984705.8028
909409.6498	381772.1396
307091.8051	436064.6698
1458443.356	941746.9228
1152737.709	1065067.172
1035201.066	776962.6295
1213721.645	1023824.034
888084.6645	819628.5394
771956.6965	389306.3696
604375.8199	496235.8442
667265.7523	665255.9719
890238.863	1493366.205
0	0
1323463.953	1369033.503
1213717.642	2004492.07
506222.7355	419792.6787
0	848588.3116
885061.6245	714214.5109
2362680.372	5608767.139
0	671943.623

228594.8802	553748.3999
3505569.411	4493595.256
0	2401217.843
0	812538.9328
0	1185035.57
59940.05037	200081.5485
902317.8531	3396460.455
203623.2687	448500.9975
432853.6504	1608749.352
37478555.49	39107447.95
58903402.29	55220400.63
664899.6154	571935.0204
922961.6242	956640.5688
2121945.76	2084616.417
5490703.139	1129314.686
1129294.935	1742265.722
3432231.099	3723828.293
1803567.495	1788204.774
4749905.12	4514810.508
2652499.511	39792.16683
14008390.07	13976720.98
2115764.228	1975308.608
0	0
325156.1685	268735.7791
398798.1001	720771.569
7001561.159	9980392.827
0	0
2319840.434	3554561.97
0	1720464.143
1988310.493	5136632.323
0	0
0	0
198584041.1	0
1162428.208	355560.0529
4178860.87	4325863.534
612604.1783	812327.2736
824007.0376	1147336.974
