# Supplementary material for: Interaction Networks Are Driven by Community-Responsive Phenotypes in a Chitin-Degrading Consortium of Soil Microbes
Source: mSystems. 2022 Sep 26;7(5):e00372-22. doi: 10.1128/msystems.00372-22 (PMC9599572; doi:10.1128/msystems.00372-22)
Supplement: TABLE S2 [file msystems.00372-22-s0008.pdf]

Supplementary Table 2. Alignment results of 16s and metatranscriptomic analyses

Sample	DNA reads	RNA reads	Aligned RNA Reads	Alignment Percentage
70 Hours A	NA	43493571	30931427	71.1
70 Hours B	NA	93592028	82581227	88.2
70 Hours C	NA	62213868	59160726	95.1
94 Hours A	102173	NA	NA	NA
94 Hours B	81848	NA	NA	NA
118 Hours A	NA	67745024	64207744	94.8
118 Hours B	NA	59462009	56797924	95.5
118 Hours C	NA	56814155	56797924	100.0
